# Quantitative Comparison of Pyranose Dehydrogenase Action on Diverse Xylooligosaccharides

**DOI:** 10.3389/fchem.2020.00011

**Published:** 2020-01-28

**Authors:** Johanna Karppi, Hongbo Zhao, Sun-Li Chong, Antti E. Koistinen, Maija Tenkanen, Emma Master

**Affiliations:** ^1^Department of Bioproducts and Biosystems, Aalto University, Espoo, Finland; ^2^Department of Food and Nutrition, University of Helsinki, Helsinki, Finland; ^3^State Key Laboratory of Subtropical Silviculture, Zhejiang A&F University, Hangzhou, China; ^4^Department of Chemical Engineering and Applied Chemistry, University of Toronto, Toronto, ON, Canada

**Keywords:** carbohydrate oxidoreductases, pyranose dehydrogenase, xylooligosaccharide, degree of oxidation, telechelic molecules

## Abstract

Pyranose dehydrogenases (PDHs; EC 1.1.99.29; AA3_2) demonstrate ability to oxidize diverse carbohydrates. Previous studies of these enzymes have also uncovered substrate-dependent regioselectivity, along with potential to introduce more than one carbonyl into carbohydrate substrates. Enzymatic oxidation of carbohydrates facilitates their further derivatization or polymerization into bio-based chemicals and materials with higher value; accordingly, PDHs that show activity on xylooligosaccharides could offer a viable approach to extract higher value from hemicelluloses that are typically fragmented during biomass processing. In this study, AbPDH1 from *Agaricus bisporus* and AmPDH1 from *Leucoagaricus meleagris* were tested using linear xylooligosaccharides, along with xylooligosaccharides substituted with either arabinofuranosyl or 4-*O*-(methyl)glucopyranosyluronic acid residues with degree of polymerization of two to five. Reaction products were characterized by HPAEC-PAD to follow substrate depletion, UPLC-MS-ELSD to quantify the multiple oxidation products, and ESI-MS^n^ to reveal oxidized positions. A versatile method based on product reduction using sodium borodeuteride, and applicable to carbohydrate oxidoreductases in general, was established to facilitate the identification and quantification of oxidized products. AbPDH1 activity toward the tested xylooligosaccharides was generally higher than that measured for AmPDH1. In both cases, activity values decreased with increasing length of the xylooligosaccharide and when using acidic rather than neutral substrates; however, AbPDH1 fully oxidized all linear xylooligosaccharides, and 60–100% of all substituted xylooligosaccharides, after 24 h under the tested reaction conditions. Oxidation of linear xylooligosaccharides mostly led to double oxidized products, whereas single oxidized products dominated in reactions containing substituted xylooligosaccharides. Notably, oxidation of specific secondary hydroxyls vs. the reducing end C-1 depended on both the enzyme and the substrate. For all substrates, however, oxidation by both AbPDH1 and AmPDH1 was clearly restricted to the reducing and non-reducing xylopyranosyl residues, where increasing the length of the xylooligosaccharide did not lead to detectable oxidation of internal xylopyranosyl substituents. This detailed analysis of AbPDH1 and AmPDH1 action on diverse xylooligosaccharides reveals an opportunity to synthesize bifunctional molecules directly from hemicellulose fragments, and to enrich for specific products through appropriate PDH selection.

## Introduction

Hemicelluloses comprise a diverse group of polysaccharides present in plant cell walls, and are thought to strengthen cell wall structures through interactions with both cellulose and lignin (Ebringerová, 2006; Scheller and Ulvskov, 2010). Xylans are the dominant hemicellulose in cell walls of both grasses and deciduous trees. In deciduous trees, the main xylan is glucuronoxylan, where the β-(1 → 4)-linked D-xylopyranose (Xyl*p*) backbone can be substituted with 4-*O*-methylated glucopyranosyluronic acid (MeGlc*p*A); the Xyl*p* backbone can also be acetylated (Ebringerová, 2006; Scheller and Ulvskov, 2010). Arabinoxylans (AXs) are the main xylans in grasses, where the Xyl*p* backbone can be substituted with L-arabinofuranose (Ara*f*). In addition to Ara*f*, arabinoglucuronoxylans found in cell walls of both grasses and coniferous trees, contain glucuronic acid and MeGlc*p*A substitutions (Scheller and Ulvskov, 2010).

So far, biocatalysts that transform hemicelluloses have mainly been studied and developed to deconstruct corresponding polysaccharides to sugars that can be fermented to fuels and chemicals (Chundawat et al., 2011). These enzymes include glycoside hydrolases (GHs), carbohydrate esterases (CEs), and auxiliary activities (AAs) that are classified by the Carbohydrate-active enzyme (CAZy) database (www.cazy.org) into multiple GH, CE, and AA families (Lombard et al., 2014). Alternatively, enzymatic functionalization of hemicelluloses, including oligosaccharides liberated during lignocellulose processing, opens new possibilities to make high value bioproducts that retain the carbon and energy stored in starting carbohydrate structures.

Carbohydrate oxidoreductases that act on hemicelluloses without cleaving them are attractive targets for further functionalization (Parikka et al., 2012). Among these are AA3 and AA7 FAD-containing oxidoreductases. Briefly, AA7 activity is restricted to the anomeric carbon of carbohydrate substrates (Huang et al., 2005; Vuong et al., 2013); the resulting lactone undergoes spontaneous ring-opening to the carboxylic acid that can be used in polymerization reactions (MacCormick et al., 2018). On the other hand, AA3 oxidoreductases are reported to oxidize the anomeric carbon and secondary hydroxyls to carboxylic acid or ketone functional groups, respectively (Giffhorn et al., 2000; Levasseur et al., 2013; Sützl et al., 2018). Based on phylogenetic analyses, AA3 sequences have been divided into four subfamilies, including activities such as cellobiose dehydrogenase, alcohol oxidase and pyranose oxidase in subfamilies 1, 3, and 4, respectively. Subfamily 2 is the most complex one of these, comprising enzymes with aryl-alcohol oxidase, aryl-alcohol dehydrogenase, glucose oxidase, glucose dehydrogenases, or pyranose dehydrogenase (PDH) activities (Levasseur et al., 2013; Sützl et al., 2018). From these, AA3_2 PDHs have been shown to oxidize several monosaccharides and some oligosaccharides through single or double oxidation and substrate dependent regioselectivity (Peterbauer and Volc, 2010; Rafighi et al., 2018), which increases their attractiveness on oligosaccharide modification.

PDHs have been found in a narrow group of litter-decomposing fungi within the fungal division Basidiomycota (Peterbauer and Volc, 2010). These extracellular enzymes contain two domains, an FAD-binding and substrate-binding domain (Tan et al., 2013). The active site contains two conserved histidines. His512 of AmPDH1 from *Leucoagaricus meleagris* acts as the main base in the first reductive half-reaction and His556 together with Gln392 and Tyr510 take part in substrate interactions that likely influence the observed substrate specific regioselectivity (Graf et al., 2013, 2015; Tan et al., 2013). After the first half-reaction, the reduced flavin is oxidized by an electron acceptor which acts as the second substrate in the reaction. Along with AmPDH1, AbPDH1 from *Agaricus bisporus* are the two most studied PDHs. AbPDH1 and AmPDH1 oxidize D-xylose to 2,3-diketo-D-xylose (Volc et al., 2000; Sedmera et al., 2006) and display slightly higher relative activity toward D-xylose compared to D-glucose (Volc et al., 2000; Sedmera et al., 2006). In addition to D-xylose, AmPDH1 was shown to oxidize xylobiose (X_2_) (Sygmund et al., 2008). PDH activity on oligosaccharides with degree of polymerization (DP) greater than two has been tested using cello- and maltooligosaccharides. Those studies confirm that AbPDH1 and AmPDH1 are both active toward cellotetraose and maltotriose (Volc et al., 1997; Peterbauer and Volc, 2010), and that AmPDH1 is also active toward maltooligosaccharides up to maltoheptaose (Tasca et al., 2007; Peterbauer and Volc, 2010; Rafighi et al., 2018). Among the characterized PDHs, AmPDH1 showed highest catalytic efficiency (k_cat_/K_m_) toward D-xylose (Sygmund et al., 2012), whereas AbPDH1 showed highest catalytic efficiency toward cellobiose, maltose and lactose (Gonaus et al., 2016).

PDH action toward xylooligosaccharides common to hardwood and agricultural resources has not been investigated. Herein, AbPDH1 and AmPDH1 were directly compared in terms of activity toward linear and substituted xylooligosaccharides, and ability to oxidize corresponding substrates at more than one position to create a new class of telechelic building blocks. The analysis of corresponding oligosaccharide products is complicated by the multiple hydroxyl groups that could potentially be oxidized. Moreover, the oxidized products are inherently unstable in water because they can further transform into various end products. For example, oxidation of the anomeric carbon leads to a lactone which spontaneously hydrolyzes to the carboxylic acid in water (Vuong et al., 2013). Furthermore, secondary hydroxyls that are oxidized to ketones and primary hydroxyls that are oxidized to aldehydes, exist primarily as hydrates (geminal diols) in water (Volc et al., 2002; Andberg et al., 2017). In this study, a new analytical method was developed to simplify and clarify the analysis of oxidized xylooligosaccharides utilizing deuterium to label the oxidized position. By following the deuterated residues by HILIC-MS-ELSD and ESI-MS^n^, the oxidation positions were identified and the proportion of each reaction product was quantified. Both AbPDH1 and AmPDH1 were shown to oxidize X_2_, xylotriose (X_3_) and xylotetraose (X_4_) with and without Ara*f* substitution; however, activity toward acidic glucuronoxylooligosaccharides was 10-times lower than with neutral substrates.

## Materials and Methods

### Materials

Below mentioned growth medium chemicals, yeast extract, yeast nitrogen base, and peptone were purchased from Lab M Ltd. (UK). Salts and vitamins were obtained from Sigma-Aldrich or Merck (Germany). Neutral substrates X_2_, X_3_, X_4_, 3^2^-α-L-arabinofuranosyl-xylobiose (A^3^X), 2^3^-α-L-arabinofuranosyl-xylotriose (A^2^XX), 3^3^-α-L-arabinofuranosyl-xylotetraose (XA^3^XX) were purchased from Megazyme (UK). 2^3^-(4-*O*-methyl-α-D-glucuronyl)-xylotriose (U^4m2^XX) and 2^3^-(4-*O*-methyl-α-D-glucuronyl)-xylotetraose (XU^4m2^XX) substrates were prepared as in Koutaniemi et al. (2012) by Dr. T. Vuong, University of Toronto and Dr. S. Koutaniemi, University of Helsinki and kindly provided to the study. The commercial laccase from *Trametes versicolor* (Sigma-Aldrich, Germany) was used in oxidation reactions (described below) to recycle 1,4-benzoquinone (BQ; Sigma-Aldrich, Germany) electron acceptor.

### PDH Production and Purification

*Agaricus bisporus* and *Agaricus meleagris* pyranose dehydrogenases (AbPDH1 and AmPDH1; pyranose:acceptor oxidoreductase, EC 1.1.99.29, CAZy family AA3_2) were expressed in *Pichia pastoris* strain KM71H. Codon optimized genes encoding AbPDH1 and AmPDH1 amino acid sequences (AAW92124 and AAW82997, respectively) were obtained as subcloned in pPICZB plasmids with C-terminal 6 x His tag (GenScript, New Jersey, USA). PDHs were produced in eight 2 l shake-flasks each containing 250 ml of medium. Precultures were grown over night in buffered glycerol-complex medium [BMGY; 100 mM potassium phosphate buffer, pH 6.0, 2% (w/v) peptone, 1% (w/v) yeast extract, 1.34% (w/v) yeast nitrogen base, 4 × 10^−5^% (w/v) biotin, 1% (v/v) glycerol] at 30°C and 220 rpm. Cells were then transferred to methanol-complex medium (BMMY) containing 0.5% (v/v) methanol instead of glycerol. Methanol was added to 0.5% (v/v) every 24 h and induction was continued 4 d at 25°C and 220 rpm. After the induction, culture supernatants were recovered and filtered, and the secreted recombinant proteins were purified by affinity chromatography; AbPDH1 was further purified by anion exchange and size exclusion (Figure S2). Final protein concentrations were measured using the Bradford method (Bio-Rad Laboratories, US); purified proteins were then aliquoted and stored in −80°C.

### Initial Activity Measurements

To select the optimal conditions for the xylooligosaccharide oxidation, the activity of AbPDH1 and AmPDH1 was screened at 30°C with 25 mM substrate (D-xylose or D-glucose) and 5 mM BQ in 50 mM sodium acetate, 50 mM ammonium acetate (at pH values 3.0–5.5), and in 50 mM sodium phosphate (at pH values 6.0–7.0) buffers. Reduction of BQ (ε_abs290nm_ = 2.24 mM^−1^ cm^−1^) in 250 μl reaction was followed at 290 nm; all reactions were performed in triplicate.

Enzyme loading for PDH conversion of xylooligosaccharides was based on activity units determined in 50 mM ammonium acetate buffer (pH 5.5). PDH activity was measured using 25 mM D-xylose and 5 mM BQ similarly as mentioned above. Laccase activity was measured using 5 mM hydroquinone (HQ, Sigma Aldrich, Germany), and oxidation of HQ (ε_abs249_ = 17.25 mM^−1^ cm^−1^) in 250 μl reaction was followed at 249 nm.

Initial AbPDH1 and AmPDH1 activity toward xylooligosaccharides was measured using 10 mM neutral or 2 mM of acidic substrates, and 0.2 mM ferrocenium hexafluorophosphate (Fc+, Sigma Aldrich, Germany) as the electron acceptor. Reactions (100 μl) were performed at 30°C in 50 mM sodium phosphate buffer (pH 7.5), and the reduction of the Fc+ ion (ε_abs250nm_ = 10.6 mM^−1^ cm^−1^) was measured for up to 1 h at 250 nm. All the activity measurements were followed using an Eon plate reader (BioTek, USA).

### Enzymatic Conversion of Xylooligosaccharides

Reactions (30–400 μl reaction volume in 1.5 ml Eppendorf tubes) were performed at 30°C for up to 48 h with shaking (500 rpm) in 10 mM ammonium-acetate buffer (pH 5.5) containing 1 mM BQ as the electron acceptor, and 5 mM of neutral xylooligosaccharides or 4 mM of acidic xylooligosaccharides (Table 1). Enzyme loadings for reactions containing neutral xylooligosaccharides were 50 mU of AbPDH or AmPDH, and 50 mU of *T. versicolor* laccase. Enzyme loadings for reactions containing acidic xylooligosaccharides were 800 mU of AbPDH1 or AmPDH1, and 800 mU of *T. versicolor* laccase. Time course sampling (25 or 10 μl) for the HPAEC-PAD analyses was done at 1, 4, and 8 h for the neutral substrates and 24 h for acidic substrates. Total reaction time was 24 or 48 h. Oxygen availability was not controlled during the reactions. Reactions were stopped by filtrating the samples through 10 kDa cut off Vivaspin 500 spin columns (Sartorius, Germany) and then kept frozen at −80°C before analysis by High-Performance Anion-Exchange Chromatography Coupled with Pulsed Electrochemical Detection (HPAEC-PAD) and mass spectrometry as described below.

**Table 1:** Structures of xylooligosaccharides and oxidation reaction details.

**Substrate**	**Substrate** ** (mM)**	**PDH** ** (U/ml)**	**Reaction^a^** ** time (h)**
Xylobiose (X_2_) 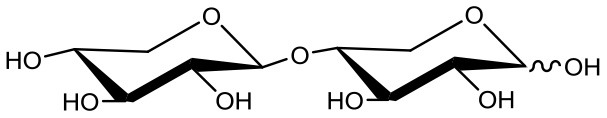	5	0.2	Up to 24
Xylotriose (X_3_) 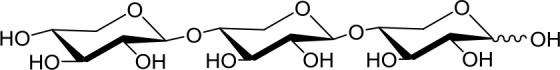	5	0.2	Up to 24
Xylotetraose (X_4_) 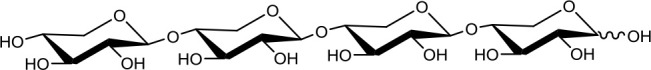	5	0.2	Up to 24
3^2^-α-L-arabinofuranosyl-xylobiose (A^3^X) 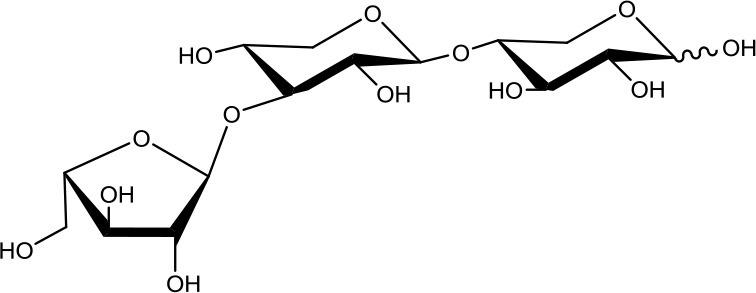	5	0.2	Up to 24
2^3^-α-L-arabinofuranosyl-xylotriose (A^2^XX) 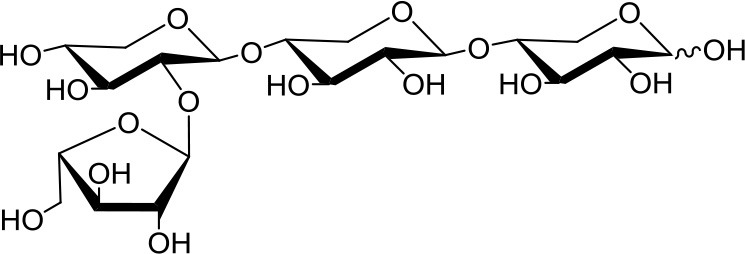	5	0.2	Up to 24
3^3^-α-L-arabinofuranosyl-xylotetraose (XA^3^XX) 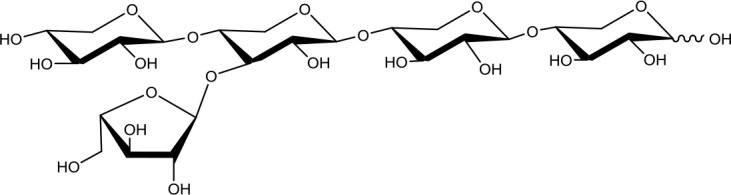	5	0.2	Up to 24
2^3^-(4-*O*-methyl-α-D-glucuronyl)-xylotriose (U^4m2^XX) 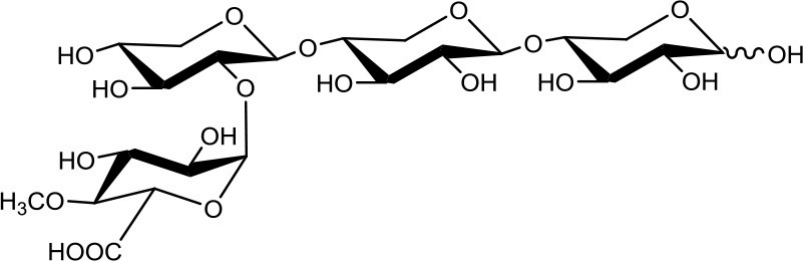	4 4	0.2 2	Up to 48 Up to 24
2^3^-(4-*O*-methyl-α-D-glucuronyl)-xylotetraose (XU^4m2^XX) 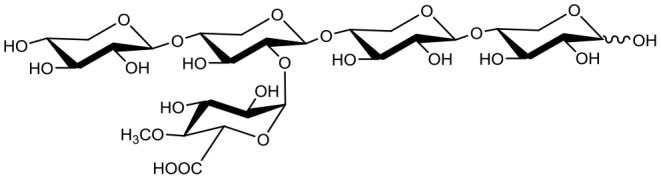	4 4	0.2 2	Up to 48 Up to 24

a *All the reactions contained 0.2 U/ml of laccase and 1 mM BQ as electron acceptor*.

### Quantification of Substrate Depletion by HPAEC-PAD

Products of reactions described above (section *Enzymatic Conversion of Xylooligosaccharides*) were diluted in ultrapure water to 50–100 ppm concentrations depending on the sensitivity of substrate detection by HPAEC-PAD. Substrate depletion over the course of the reaction was followed using a DionexTM-5000^+^ system equipped with a Dionex^TM^ CarboPac^TM^ PA1 IC column and corresponding precolumn (Thermo Scientific, USA). The samples were eluted at 1 ml/min with eluent A (0.1 M NaOH) in a linear gradient toward an increasing proportion of eluent B (1 M NaOAc in 0.1 M NaOH). The gradient reached 21.7% B at 20 min after injection, 38% B at 25 min and 100% B at 27 min after which it was kept at 100% for 7 min. Data were analyzed using the Thermo Scientific Dionex Chromeleon 7 Chromatography Data System (version 7.2 SR4, Thermo Fisher Scientific). Corresponding xylooligosaccharides with three known concentrations were used to create a standard curve for each run. Integrated peak areas of the enzyme treated xylooligosaccharides were compared against the corresponding control reactions without the PDH enzyme.

### Mass Spectrometric Analysis of Oxidized Products by Direct Infusion

A 5 μl sample from 24 h reactions described above (section *Enzymatic Conversion of Xylooligosaccharides*) was mixed with 5 μl of 10 mg/ml NH_4_Cl and 500 μl 50% acetonitrile (ACN) in water. Each sample solution was then introduced into Quadruple Time-of-flight (Q-Tof) mass spectrometry with an ESI source (SYNAPT G2-Si, Waters, MA, USA) at a flow rate of 5 μl/min. The capillary was set to 3 kV; source temperature to 80°C and desolvation temperature to 150°C. The cone gas was 100 l/h and desolvation gas was 600 l/h with nebulizer set at 6.5 bar. The analysis was done in negative mode and the ions were collected in *m/z* range of 50–800. The products of acidic xylooligosaccharides oxidized by 2 U/ml PDH for 24 h were analyzed directly by ESI-Q-Tof-MS without the addition of NH_4_Cl.

### Identification of Oxidized Positions Through Fragmentation Analysis of Products After NaBD_4_ Reduction

The multiple potential oxidations and reactivity of oxidized products in water complicate the interpretation of MS spectra. Therefore, after confirming the production of oxidized xylooligosaccharides by direct infusion mass spectrometry, reaction products were reduced using NaBD_4_ to facilitate their identification. Specifically, NaBD_4_ was added to the 24 h reactions described above (section *Enzymatic Conversion of Xylooligosaccharides*) at 3 mol equivalent NaBD_4_ per mole of substrate in the initial reaction; the resulting solution was then stirred overnight to reduce the carbonyls back to hydroxyls.

After reduction, samples were desalted using Porous Graphitic Carbon (PGC) columns (Hyper carb PGC 50 mg, Thermo Fisher, MA, USA). Briefly, a column was pre-washed with 0.1% trifluoroacetic acid (TFA) 80% ACN and water before loading a sample. The impurities were washed away with Milli Q water and the reaction products were eluted with 50% ACN. The purified samples were then lyophilized, dissolved in 200 μl MilliQ-water, and diluted to 10 μg/ml using 50% ACN, after which ammonium chloride (10 mg/ml) was added to a final concentration of 40 μg /ml. The negative ion MS and MS^n^ spectra were obtained by direction infusion of the solutions to the Finnigan LXQ Ion Trap (IT) mass spectrometer equipped with ESI source (Thermo Fisher, MA, USA) at a flow rate of 5 μl/min. The parameters were automatically tuned by the instrument based on X_3_. The collision energy for fragmentation was optimized based on each substrate.

X_2_ and its oxidized products after reduction were not successfully purified by the PGC column. Instead, they were analyzed by Acquity Ultra High Performance Liquid Chromatography (UPLC) coupled to an ESI-Q-Tof mass spectrometer (Waters, MA. USA) as described in Figure S12.

### Quantification of Oxidized Products by UPLC-ESI-Q-Tof-MS and UPLC Evaporative Light Scattering Detection (ELSD)

The reaction products after NaBD_4_ reduction and PGC purification were quantified using an Acquity UPLC coupled with ELSD (Waters, MA, USA). A 1.7 μm, 2.1^*^150 mm Acquity UPLC BEH Amide column (Waters, MA, USA) was used to separate the reaction products. The mobile phases were (A) ACN with 0.1% ammonium hydroxide and (B) 20% ACN with 0.1% ammonium hydroxide. The elution gradient was as follows: from 96% ACN to 50% ACN in 10 min, isocratic (50% ACN) for 2 min, back to 96% ACN in 0.01 min, and 18 min re-equilibrium in initial conditions. The flow was at 250 μl/min with column temperature at 35°C. The ELSD drift tube temperature was set to 40°C and the gain to 200. The nebulizer was set to cooling and pressure to 40 psi. Reaction products were diluted with pure ACN to ~3,000 ng of reaction products per 7 μl of injected sample. External standards were made by reducing and purifying the pure substrates using the PGC column as described in section *Identification of Oxidized Positions Through Fragmentation Analysis of Products After NaBD*_4_ *Reduction*. Standard curves were made by injecting 200–3,750 ng of each reduced substrate for each injection. The Acquity UPLC coupled with ESI-Q-Tof-MS was used to identify the peaks in ELSD chromatogram. The parameters for ESI-Q-Tof were set to the same as mentioned in section *Mass Spectrometric Analysis of Oxidized Products by Direct Infusion*, except that the desolvation temperature was set to 400°C. Quantitative analysis of the reaction products using the UPLC-ELSD and mass spectra collected in section *Identification of Oxidized Positions Through Fragmentation Analysis of Products After NaBD*_4_ *Reduction* was then achieved as described below (section *Quantitative Interpretation of Mass Spectra*).

### Quantitative Interpretation of Mass Spectra

Following NaBD_4_ reduction of oxidized xylooligosaccharides, the same *m/z* value can be obtained for isotopic non-oxidized xylooligosaccharides and specific oxidized products. For example, the monoisotopic mass of X_3_ has an *m/z* of 452, and the naturally present isotopic chloride, carbon, hydrogen and oxygen generate *m/z* values of 453 and 454. At the same time, single oxidation of a secondary hydroxyl in X_3_ followed by NaBD_4_ reduction will also generate a peak at *m/z* 453, and double oxidation of secondary hydroxyls in X_3_ followed by NaBD_4_ reduction will generate a peak at *m/z* 454 (Figure S1). Therefore, the following system of equations (Equations 1–4) was established to calculate the percent of single oxidized and double oxidized products, taking into account the abundance of naturally present isotopic substrate:

(1) $$\begin{array}{l} \text{Abundance of isotopic }m/z\text{ for a given reduced oligosaccharide}\\ =\text{abundance of non-oxidized oligosaccharide}\\ +\text{abundance of single oxidized oligosaccharide}\\ +\text{abundance of double oxidized oligosaccharide}\\ =a+\left(b-a\times r1÷100\right)\\ +\left\{c-a\times r2÷100-\left(b-a\times r1÷100\right)\times r1÷100\right\} \end{array}$$

where a, b, and c are the abundance of *m/z* values corresponding to non-oxidized, single oxidized, and double oxidized oligosaccharides in reactions following enzymatic oxidation (e.g., *m/z* 452, 453, and 454, respectively, for X_3_); and r1 and r2 are the abundances of corresponding *m/z* values naturally present the isotopic substrate.

Therefore:

(2) $$\begin{array}{l} \text{Non}-\text{oxidation}\%=\;100\%\;-\text{single oxidation}\%\\ \;-\text{ double oxidation}\% \end{array}$$

where:

(3) $$\begin{array}{l} \text{Single oxidation}\%\\ =\frac{\text{Abundance of single oxidized oligosaccharide}}{\text{Abundance of isotopic }m/z\text{ for a given reduced oligosaccharide}}\\ \;\times 100\% \end{array}$$

(4) $$\begin{array}{l} \text{Double oxidation}\%\\ =\frac{\text{Abundance of double oxidized oligosaccharide}}{\text{Abundance of isotopic }m/z\text{ for a given reduced oligosaccharide}}\\ \;\times 100\% \end{array}$$

When an oligosaccharide is oxidized at the reducing end C-1, the reaction product containing the carboxyl acid forms an anionic, deprotonated species. For example, X_3_ oxidized at the C-1 position will have an *m/z* of 429. Herein, the theoretical isotopic distribution of C-1 oxidized oligosaccharides was generated by Masslynx V4.1. When additional oxidations take place at the secondary hydroxyls, the deuterium label would increase the corresponding *m/z* value by a corresponding number of Daltons. Therefore, Equations (1)–(4) can be extended by Equations (5)–(8), to calculate the extent of oxidized products with at least one oxidation at the reducing end C-1:

(5) $$\begin{array}{l} \text{Abundance of isotopic m}/\text{z for a given oligosaccharide }\\ \text{oxidized at the reducing end C-}1\\ =\text{abundance of C-}1\text{ oxidized oligosaccharide}\\ +\text{ abundance of double oxidized oligosaccharide}\\ +\text{ abundance }\mathrm{of}\;\mathrm{triple}\text{ oxidized oligosaccharide}\\ =\;a^{\prime }+\;\left(b\prime -a\prime \times r1^{\prime }÷100\right)\\ +\left\{c\prime -a\prime \times r2^{\prime }÷100-\left(b^{\prime }-a^{\prime }\times r1^{\prime }÷100\right)\times r1^{\prime }÷100\;\right\} \end{array}$$

where a′, b′, and c′ are the abundance of *m/z* values corresponding to C-1 oxidized, C-1 oxidized + single oxidation at a secondary hydroxyl, and C-1 oxidized + double oxidation at secondary hydroxyls (e.g., *m/z* 429, 430, and 431, respectively, for X_3_); and r1′ and r2′ are the relative abundance of theoretically generated isotopic oligosaccharide with one oxidation at reducing end C-1.

In this case, the double oxidation consists of one reducing end C-1 oxidation and one secondary hydroxyl oxidation, and a the triple oxidation consists of one reducing end C-1 oxidation and two secondary hydroxyl oxidation.

Therefore:

(6) $$\begin{array}{l} \text{Reducing end C-}1\text{ oxidation}\%\;=\;100\%\;-\text{double oxidation}\%\\ \;-\text{triple oxidation}\% \end{array}$$

where:

(7) $$\begin{array}{l} \text{Double oxidation}\%\\ =\frac{\begin{array}{c} \text{ Abundance of double oxidized }X_{3} \end{array}}{\begin{array}{c} \text{Abundance of isotopic m}/\text{z for a given }\\ \text{oligosaccharideoxidized at the reducing end C-}1 \end{array}}\times 100\% \end{array}$$

(8) $$\begin{array}{l} \text{Triple oxidation}\%\\ =\frac{\begin{array}{c} \text{Abundance of triple oxidized }X_{3} \end{array}}{\begin{array}{c} \text{Abundance of isotopic m}/\text{z for a given oligosaccharide}\\ \text{oxidized at the reducing end C-}1\; \end{array}}\times 100\% \end{array}$$

See Figure S1 for example calculations.

## Results

### Initial Rates of PDH Action on Xylooligosaccharides

**Figure 1:**
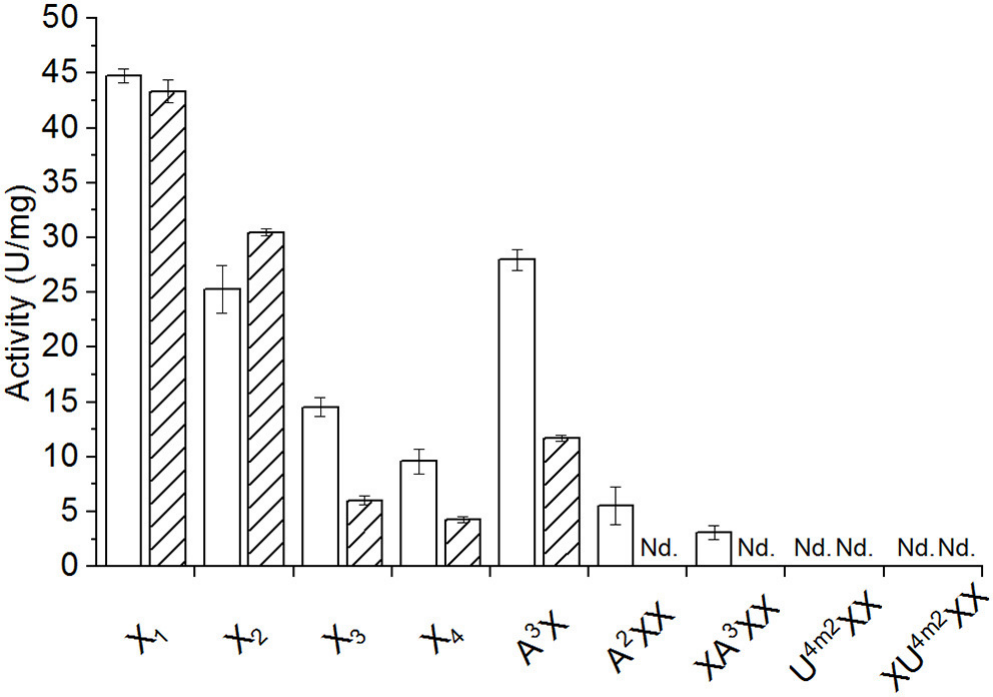
Activity of AbPDH1 (white) and AmPDH1 (with diagonal stripes) on 10 mM of neutral or 2 mM of acidic xylooligosaccharides at pH 7.5 using Fc+ ion as electron acceptor. X_1_, xylose; X_2_, xylobiose; X_3_, xylotriose; X_4_, xylotetraose; A^3^X, 3^2^-α-L-arabinofuranosyl-xylobiose; A^2^XX, 2^3^-α-L-arabinofuranosyl-xylotriose; XA^2^XX, 3^3^-α-L-arabinofuranosyl-xylotetraose; U^4m2^XX, 2^3^-(4-*O*-methyl-α-D-glucuronyl)-xylotriose; XU^4m2^XX, 2^3^-(4-*O*-methyl-α-D-glucuronyl)-xylotetraose. Error bars represent standard deviation of three replicate reactions. Reactions with U^4m2^XX and XU^4m2^XX were done in duplicates. Nd., no activity detected under these conditions after 24 h.

The yield of recombinantly produced and purified AbPDH1 and AmPDH1 was 1.65 and 11.5 mg/l, respectively, where over 90% purity was reached for both enzymes (Figure S2). The activity and pH optima of each PDH was comparable to previous studies when using glucose as the substrate (Table S1). Activity on xylose and xylooligosaccharides was then measured using Fc+ ion as the electron acceptor, given the particular sensitivity of the corresponding assay (Sygmund et al., 2008). Both AbPDH1 and AmPDH1 were active on 10 mM X-X_4_ and A^3^X; furthermore, AbPDH1 was distinguished by its additional activity on A^2^XX and XA^3^XX under the conditions used (Figure 1).

Activity of both enzymes on xylose (43–45 U/mg; Figure 1) were similar to those previously reported for the native AmPDH1 (39 U/mg; Sygmund et al., 2008). Overall, AbPDH1 had higher activities toward X_3_, X_4_, and A^3^X compared to corresponding activity of AmPDH1. Notably, the activity of AbPDH1 toward A^3^X was comparable to unsubstituted X_2_, and AbPDH1 activity toward A^2^XX and XA^3^XX was ~40 and 55% of the corresponding unsubstituted X_3_ and X_4_ substrates. By contrast, the activity of AmPDH1 on A^3^X was only 38% of its activity on X_2_ and initial activity values could not be obtained for AmPDH1 oxidation of A^2^XX or XA^3^XX under these conditions. Similarly, initial activities were not obtained for AbPDH1 and AmPDH1 toward U^4m2^XX or XU^4m2^XX; however, oxidation of all tested xylooligosaccharides by AbPDH1 and AmPDH1 was observed after reaction optimization and prolonged incubation time, as described below.

### Measuring Xylooligosaccharide Conversion by AbPDH1 and AmPDH1

To maximize xylooligosaccharide oxidation by AmPDH1 and AbPDH1, the Fc+ electron acceptor used to measure initial rates was replaced with BQ that can be regenerated using laccase (Baminger et al., 2001). In an effort to optimize PDH and laccase activities, glucose and xylose oxidation by AmPDH1 and AbPDH1 was tested at pH values ranging from pH 3.0 to 7.0 (Figure S3). Based on these analyses, subsequent reactions were performed at pH 5.5. Notably, the addition of laccase clearly increased substrate conversions by both enzymes (Figure S4), where up to 85% depletion of xylose (50 mM starting concentration) was reached in 24 h using 5 mM BQ.

**Figure 2:**
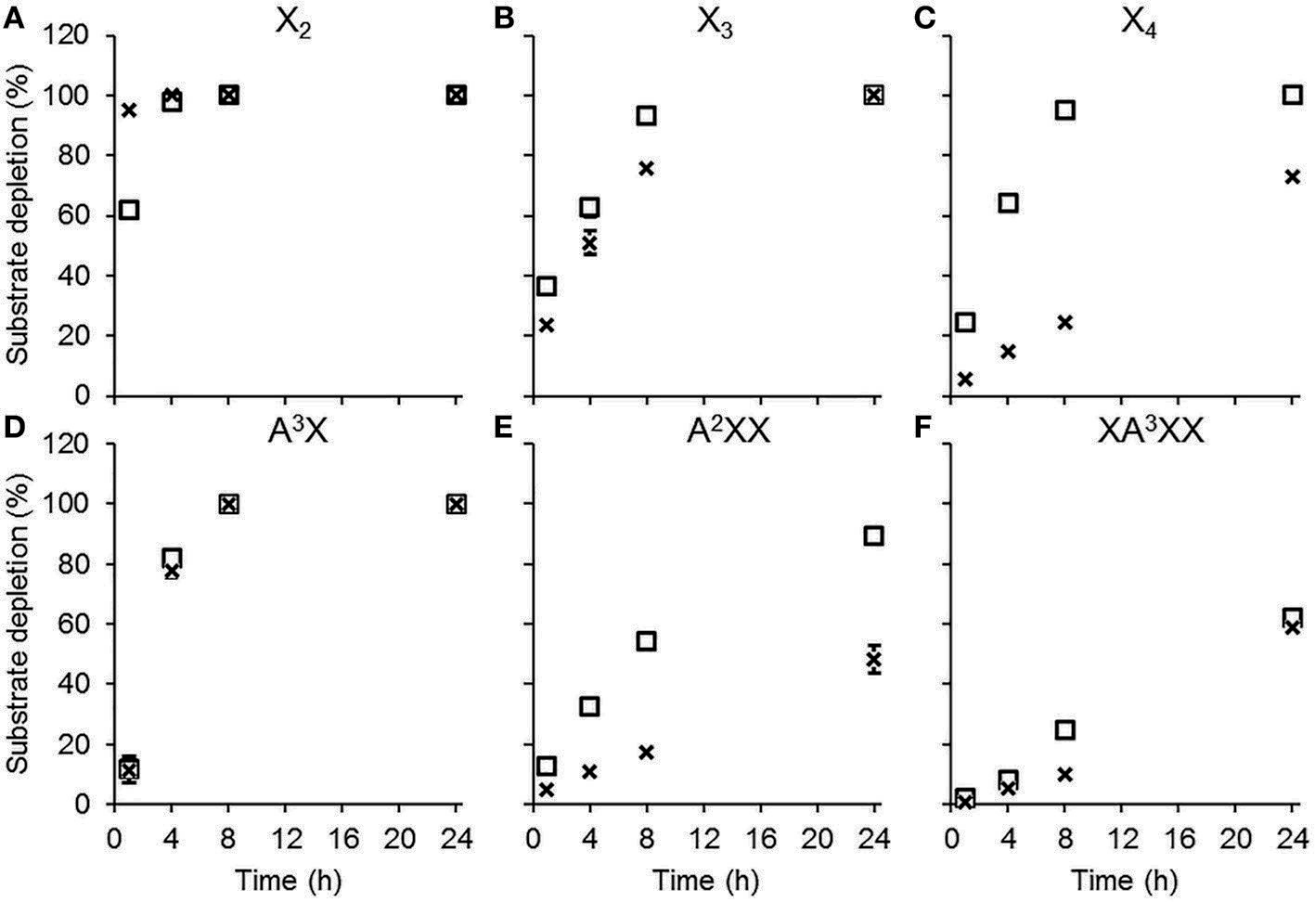
Substrate depletion by AbPDH1 (square) and AmPDH1 (cross) followed by HPAEC-PAD. Substrates: **(A)** xylobiose, X_2_, **(B)** xylotriose, X_3_, **(C)** xylotetraose, X_4_, **(D)** 3^2^-α-L-arabinofuranosyl-xylobiose A^3^X, **(E)** 2^3^-α-L-arabinofuranosyl-xylotriose, A^2^XX, and **(F)** 3^3^-α-L-arabinofuranosyl-xylotetraose XA^3^XX. Depletion of 5 mM substrate using 0.2 U/ml PDHs, 1 mM BQ and 0.2 U/ml laccase was compared to the control reaction containing all reaction components except PDH. Error bars show the data range of duplicate reactions. Error bars are not visible when the data range fits inside the drawn data point.

Both AbPDH1 and AmPDH1 fully depleted X_2_, X_3_, and A^3^X after 24 h; AbPDH1 also fully depleted X_4_ after 24 h (Figure 2). With the exception of X_2_, substrate depletion was more rapid in reactions containing AbPDH1 than AmPDH1 (Figure 2). Differences in substrate conversion were most apparent after 8 h, where X_3_ and X_4_ conversion by AbPDH1 was 1.2 and 3.9 times higher than by AmPDH1, respectively (Figures 2B,C).

When testing AbPDH1 and AmPDH1 activity over 24 h on A^3^X, A^2^XX, and XA^3^XX, minor α-arabinofuranosidase activity was detected in the commercial laccase. Specifically, after 24 h in reactions containing laccase alone, 7.5, 11.5, and 20% of Ara*f* was hydrolyzed from A^3^X, A^2^XX, and XA^3^XX, respectively (Figures S5–S7). This reduction of substrate by hydrolysis was taken into account by comparing the substrate depletion in PDH reactions to corresponding control reactions containing laccase alone. Whereas, both AbPDH1 and AmPDH1 were able to fully deplete A^3^X by 8 h (Figure 2D), A^2^XX conversion by AbPDH1 was 1.6 times higher than AmPDH1 after 24 h (Figure 2E), and both AbPDH1 and AmPDH1 transformed ~60% of XA^3^XX after 24 h (Figure 2F).

When using the same enzyme loading (0.2 U/ml) as for neutral xylooligosaccharides, only AbPDH1 showed clear conversion of both U^4m2^XX and XU^4m2^XX (Figure S8). Detectable conversion of U^4m2^XX and XU^4m2^XX by AmPDH1 was only observed after increasing the enzyme loading 10 times (to 2 U/ml). When comparing AbPDH1 and AmPDH1 after 24 h at the higher enzyme loading, AbPDH1 depleted nearly 100% of both U^4m2^XX and XU^4m2^XX whereas substrate depletion by AmPDH1 was ~30 and 20% for U^4m2^XX and XU^4m2^XX, respectively (Figure S8).

### Direct Infusion ESI-Q-Tof Mass Spectrometry Confirms the Formation of Oxidized Xylooligosaccharides

Direct infusion ESI-Q-Tof analyses confirmed that all the tested substrates were oxidized by both AbPDH1 and AmPDH1. The C-1 oxidation products were negatively charged and detected in their anionic form, while neutral oligosaccharides were detected as chlorine adducts. X_2_, X_3_, X_4_, A^3^X, A^2^XX, XA^3^XX gave peaks at mass-to-charge ratios (*m/z*) 317, 449, 581, 449, 581, and 713, respectively (Figures S9, S10). Consistent with the HPAEC-PAD analyses summarized above, low levels of products with a loss of pentose (132 Da) were detected in reactions containing Ara*f* substituted xylooligosaccharides, resulting from α-arabinofuranosidase side activity in the commercial laccase preparation. U^4m2^XX and XU^4m2^XX were detected deprotonated [M-H]^−^ with peaks at *m/z* 603 and 735, respectively (Figure S11).

Both AbPDH1 and AmPDH1 completely consumed X_2_ and X_3_ after 24 h treatment. By contrast, varying amounts of residual substrate could be detected for X_4_ and all substituted xylooligosaccharides. AbPDH1 and AmPDH1 treatment of X_2_, A^3^X, and XA^3^XX resulted in similar MS spectra and thus end products; however, clear differences in product profiles were seen for the other tested substrates. For example, highest peaks following X_3_ oxidation by AbPDH1 and AmPDH1 were at *m/z* 463 and 427, respectively; and highest peaks following X_4_ oxidation were at *m/z* 595/597 and 561, respectively (Figure S9). Considering substituted xylooligosaccharides, a peak at *m/z* 597 represented the highest product peak from A^2^XX following 24 h treatment with AbPDH1, whereas the most abundant peak after 24 h AmPDH1 treatment was the non-oxidized A^2^XX (*m/z* 581; Figure S10). Consistent with the greater oxidation of neutral xylooligosaccharides by AbPDH1 compared to AmPDH1, the ESI-Q-Tof analyses confirmed significant oxidation of U^4m2^XX and XU^4m2^XX by the higher dose of AbPDH1, resulting in peaks at *m/z* 619 and 751, respectively. By contrast, AmPDH1 oxidized U^4m2^XX and XU^4m2^XX to a small percentage (Figure S11). Given the higher oxidation of neutral substrates by both enzymes, the corresponding products were analyzed in greater detail.

### Qualitative Comparison of Oxidized Xylooligosaccharides Generated by AbPDH1 and AmPDH1

Due to the multiple potential oxidations and reactivity of the oxidized products in water, MS spectra interpretation is challenging. Taking the AbPDH1 oxidized X_3_ as an example (Figure S1C), the main peak *m/z* 463 is expectedly representing a chlorine adduct of a double oxidized product with two carbonyl groups of which the other one has reacted with water to a hydrate (compound A in Table S2). Peak *m/z* 481 is presumably the chorine adduct of the same double oxidized product in which both carbonyls are in the hydrate form. The second most abundant peak *m/z* 427 is most probably the double oxidized product carrying carboxylic acid group at the reducing end C-1 and one carbonyl group (compound B in Table S2) whereas peak *m/z* 445 can represent the same product in a hydrate form. Mass to charge ratio of 445 can also represent a chlorine adduct of the original double oxidized product with two carbonyl groups. Thus, one compound that is oxidized at two secondary hydroxyls is shown as three peaks in the mass spectrum (compound A in Table S2).

**Figure 3:**
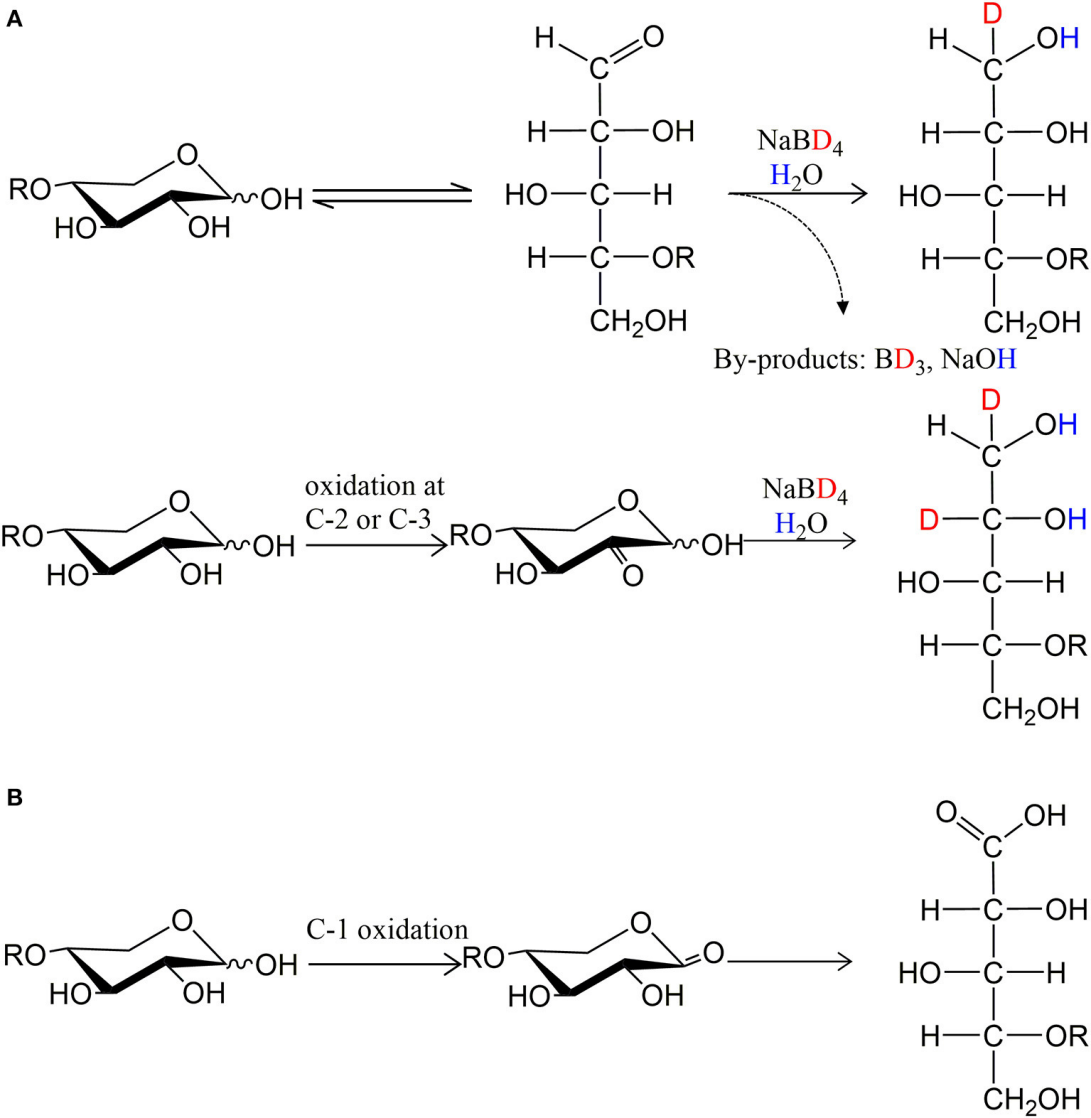
The principle of isotopic labeling with NaBD_4_ reduction. **(A)** One oxidation at secondary hydroxyls is equivalent to one Dalton difference in mass and **(B)** oxidation at C-1 hydroxyl is resistant to NaBD_4_ reduction.

To facilitate the interpretation of MS spectra, the oxidized products were treated with NaBD_4_, and analyzed by ESI-MS after purification. Thus, a PDH oxidation at secondary hydroxyl resulting in a ketone is equivalent to one Dalton difference in mass after NaBD_4_ reduction compared to substrate (Figure 3A). Instead, the oxidation at the reducing end C-1 forms a carboxyl group, which is resistant to NaBD_4_ reduction (Figure 3B). Compared to the chloride adduct of NaBD_4_ reduced substrate, the oxidation at reducing end C-1 will result in a deprotonated *m/z* peak that is 23 Da less. Therefore, by counting the Dalton difference between the reduced oxidation products and the reduced original substrates, the number of oxidations occurring in one molecule can be determined.

After NaBD_4_ reduction, the predominant peak in mass spectra of oxidized X_2_ by both PDHs was *m/z* 322, which is 2 Da more than the control X_2_ indicating double oxidation at two secondary hydroxyls (Figure S12). The 2 Da increase in mass was also seen for oxidized X_3_. Oxidation of X_4_ by both PDHs resulted in single and double oxidized products after 24 h reaction. Notably, Ara*f* substitution inhibited multiple oxidation of corresponding substrates; A^3^X and A^2^XX were mostly single oxidized at a secondary hydroxyl while the XA^3^XX was still largely non-oxidized by both PDHs after 24 h reaction (Figure S13).

In addition to oxidizing secondary hydroxyls, both AbPDH1 and AmPDH1 oxidized the reducing end C-1 of all substrates. Moreover, mass spectra of X_2_, X_3_, and X_4_ products revealed that reducing end C-1 oxidation and secondary hydroxyl oxidations could co-exist in one molecule, resulting in final products with a mass that is 22 Da (reducing end C-1 oxidation + one secondary hydroxyl oxidation) or 21 Da (reducing end C-1 oxidation + two secondary hydroxyls oxidation) less than the control substrate (Figure S12).

Direct infusion mass spectrometry after NaBD_4_ reduction thus confirmed the ability of both AbPDH1 and AmPDH1 to oxidize xylooligosaccharides at multiple positions. Quantification of the reaction products was subsequently important to uncover the specificity of each PDH.

### Separate Quantification of Xylooligosaccharides Oxidized at Secondary Hydroxyl Positions and the Reducing End C-1

A two-stage MS-based approach was developed in this study to quantify single and multiply oxidized oligosaccharides, and as well as oxidations at secondary hydroxyls vs. the reducing end C-1 (section *Quantitative Interpretation of Mass Spectra*; see Figure S1 for example calculation).

**Figure 4:**
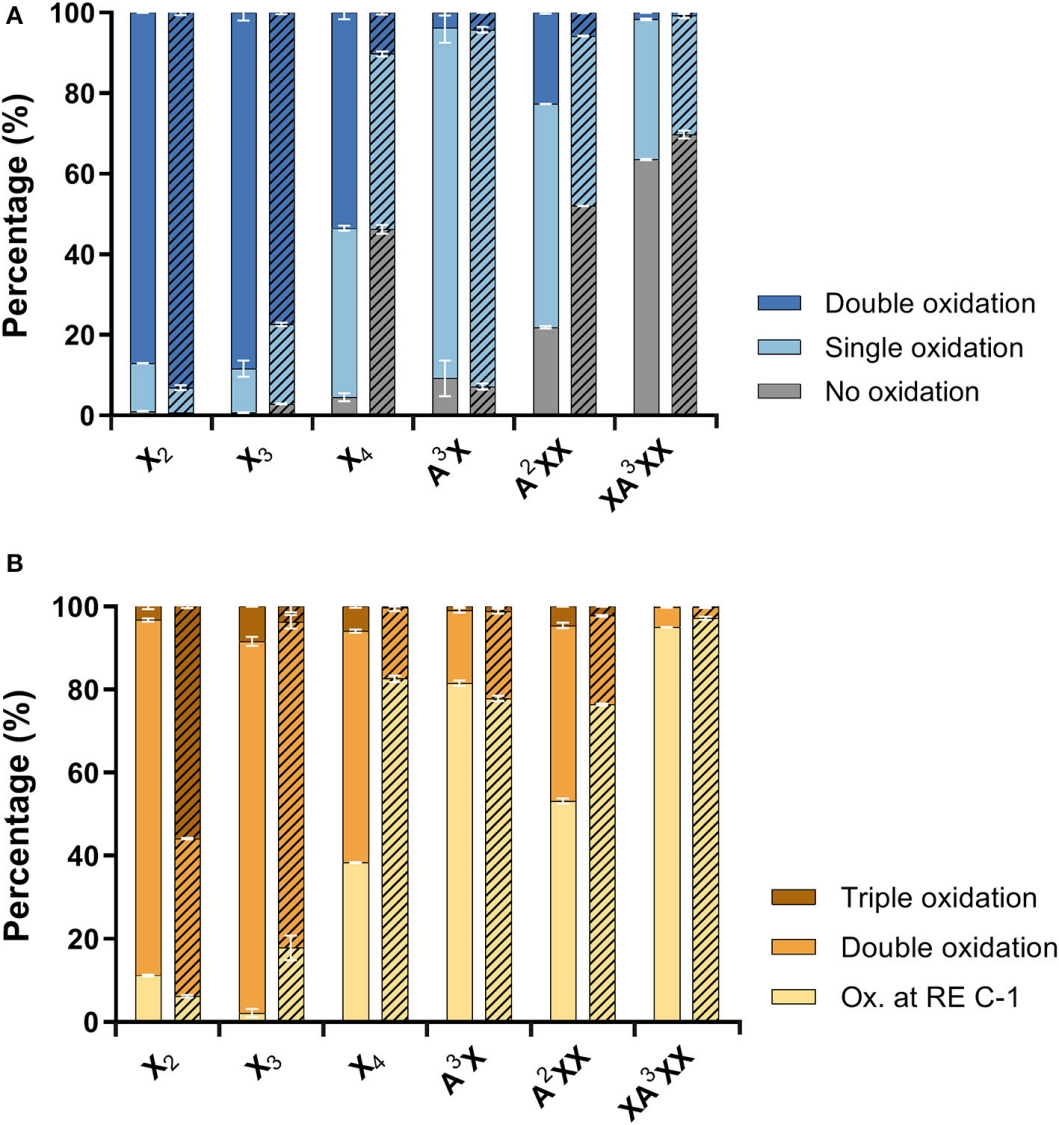
Distribution of **(A)** neutral products (unreacted substrate and oxidations at secondary hydroxyls) and **(B)** acidic products (reducing end C-1 oxidation with zero, one or two secondary hydroxyls oxidations). Reactions with AbPDH1 are indicated by solid color and reactions with AmPDH1 are indicated by diagonal stripes.

Product profiles were first calculated based on mass spectra, where different ionization intensities are obtained for neutral and acidic products, preventing the direct quantitative comparison of residual substrate products with ketone groups, and acidic products with reducing end C-1 oxidation. As a result, the extent of oxidized secondary hydroxyls and the reducing end C-1 were calculated separately (Figure 4). Overall, products with two ketones (i.e., oxidation of two secondary hydroxyls) were dominant for linear xylooligosaccharides. The only exception was AmPDH1 oxidation of X_4_, which was only partially oxidized and mainly to a single oxidized product (Figure 4A). For all linear xylooligosaccharides treated for 24 h with AbPDH1, products oxidized at the reducing end C-1 were mostly also oxidized at a secondary hydroxyl group (i.e., were double oxidized products, Figure 4B). This same pattern was observed for AmPDH1 oxidation of X_3_; however, a triple oxidized product (C-1 oxidation + two ketones) represented more than 50% of acidic products generated through AmPDH1 oxidization of X_2_. Whereas, oxidation of linear xylooligosaccharides mostly led to double oxidized products, single oxidized products dominated in reactions containing Ara*f* substituted xylooligosaccharides (Figures 4A,B).

### Total Comparative Quantification of Oxidized Xylooligosaccharides Generated by AbPDH1 and AmPDH1

**Figure 5:**
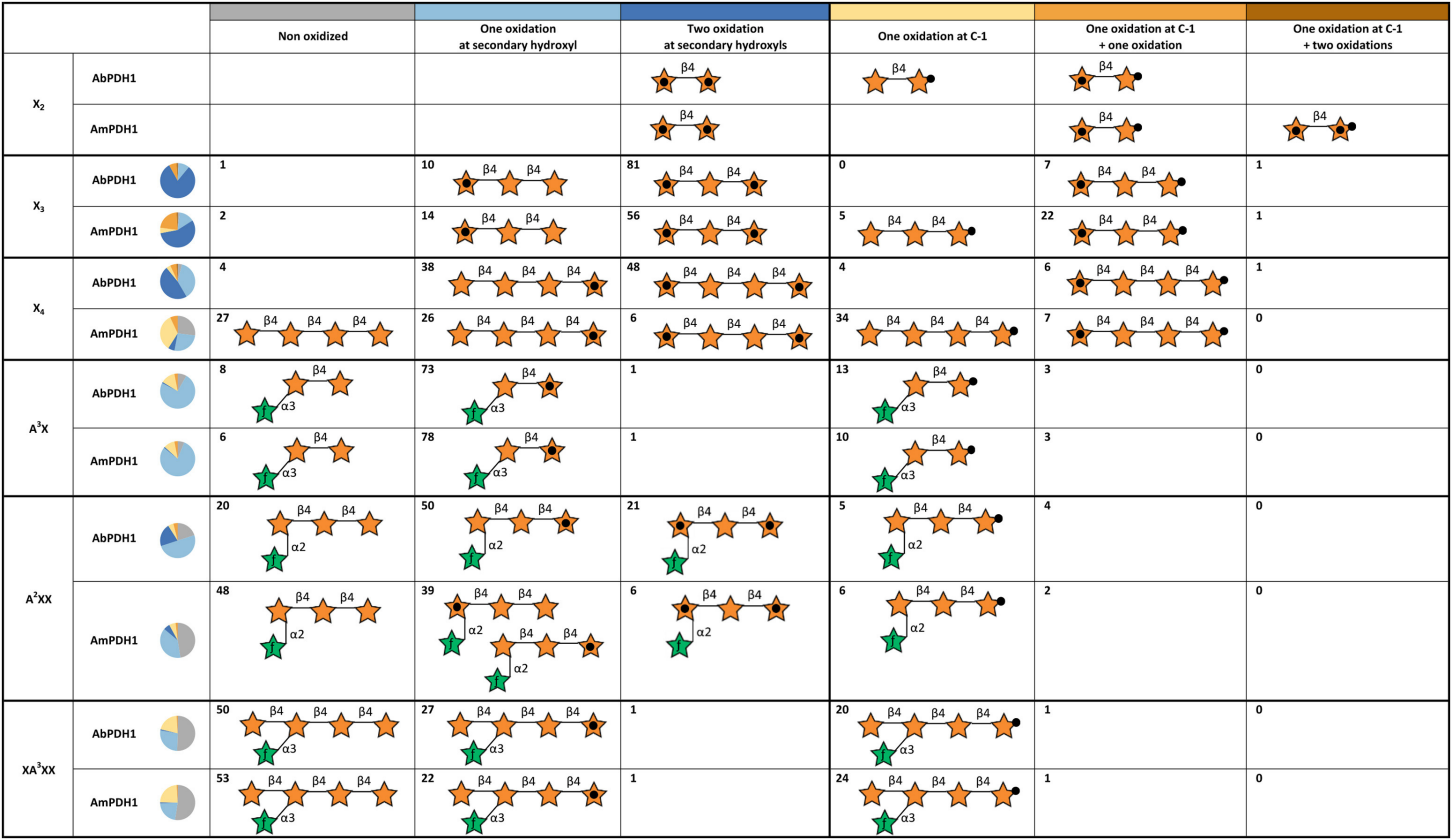
Summary of main oxidation products and oxidized positions following xylooligosaccharide treatment by AbPDH1 and AmPDH1. Representative product structures are drawn for those representings > 5% of the reaction mixture; the symbol nomenclature for glycans was adopted from Varki et al. (2015). A black dot in the center of a star represents a secondary hydroxyl oxidation, and a black dot at the tip of a star represents oxidation at the reducing end C-1. The proportion of each reaction product is shown by the number in each cell and summarized by the pie chart shown in each row.

Using hydrophilic interaction chromatography (HILIC), acidic and neutral reaction products were successfully separated and identified by MS (Figures S14, S15). The acidic products (C-1 oxidation) eluted earlier than the neutral ones and these two classes of products could be quantified after HILIC separation with on-line ELSD using external standards. The ELSD quantification was successfully done for X_3_, X_4_, A^3^X, A^2^XX, and XA^3^XX products. Due to co-elution with salts, X_2_ products could not be quantified. Six reaction species could be quantified: non-oxidized substrate, one oxidation at a secondary hydroxyl, two oxidations at secondary hydroxyls, one oxidation at the reducing end C-1, one oxidation at reducing end C-1 with one oxidation at secondary hydroxyl, and one oxidation at reducing end C-1 with two oxidations at secondary hydroxyls (Figure 5).

AbPDH1 and AmPDH1 primarily oxidized secondary hydroxyls, and to a lesser extent the anomeric C-1. The main, double oxidized derivative with two ketone groups represented 81 and 56% of X_3_ products after 24 h treatment by AbPDH1 and AmPDH1, respectively (Figure 5). AbPDH1 also mainly oxidized X_4_ at secondary hydroxyls with 38% being single ketone and 48% being double ketone products, whereas the most abundant reaction product after AmPDH1 treatment was C-1 oxidized X_4_ (34%). Dominating A^3^X products (73–78%) carried single ketone group whereas A^2^XX was primary oxidized by AbPDH1 to one (50%) and two (21%) ketone derivatives. After AmPDH1 treatment, 48% of A^2^XX remained non-oxidized and 39% of products carried single ketone group. The composition of reaction products from XA^3^XX treatments by both PDHs was rather similar, with ~50% of substrates being non-oxidized after 24 h reaction; ~25% converted to products with one ketone group, and 22% were oxidized only at the reducing end C-1 (Figure 5).

### MS Fragmentation to Determine the Oxidized Residues

**Figure 6:**
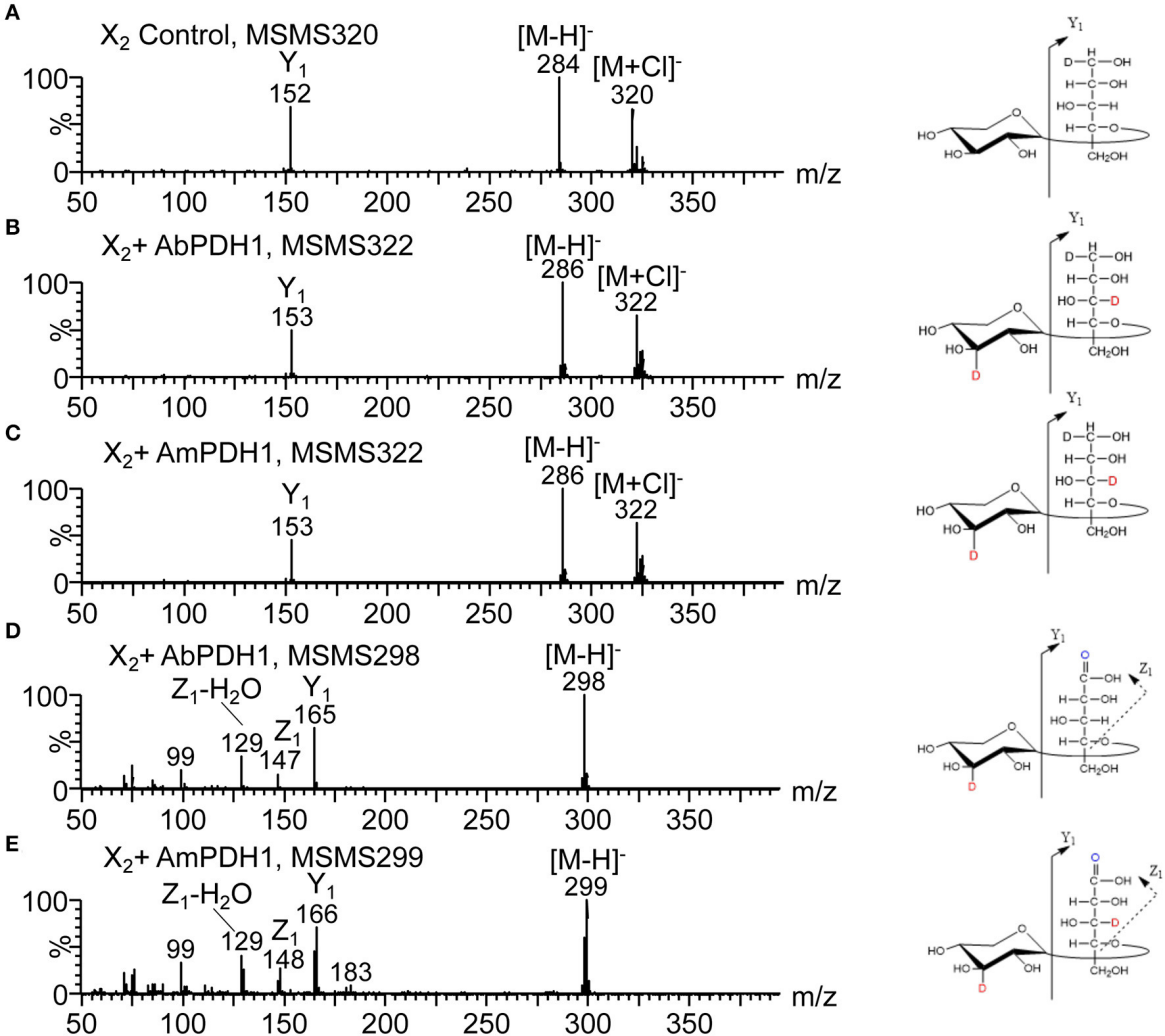
HILIC-MS/MS spectra collected in negative ion mode showing fragmentation of X_2_ after enzymatic oxidation by PDHs followed by NaBD_4_ reduction. **(A)** Negative MS/MS 320 of the X_2_ control, **(B)** negative MS/MS 322 of the X_2_ oxidized by AbPDH1, **(C)** negative MS/MS 322 of the X_2_ oxidized by AmPDH1, **(D)** negative MS/MS 298 of the X_2_ oxidized by AbPDH1, **(E)** negative MS/MS 299 of the X_2_ oxidized by AmPDH1. Product ions named according to Domon and Costello (1988). Oxidations at C-2, C-3, and C-4 are not distinguishable.

The oxidized products after reduction were analyzed using Q-Tof or ion trap by following the deuterium label in the fragment ions. For example, the MS/MS spectrum of reduced X_2_ shows the glycosidic linkage cleavage generating Y_1_ ion containing the reduced Xyl*p* residues at *m/z* 152 (Figure 6A). The molecular masses of Y_1_ ions carrying 1 Da more than the control sample (Figures 6B,C), indicate that one oxidation had taken place at the reducing Xyl*p* residue by both AbPDH1 and AmPDH1. The mass of precursor ions increased by 2 Da for oxidized samples, thus the other oxidation was at the non-reducing end Xyl*p* residue. The C-1 oxidized X_2_ would have *m/z* 297 and generate the Y_1_ ion at *m/z* 165 (data not shown). The *m/z* 298 ion produced the Y_1_ ion also at *m/z* 165 (Figure 6D), suggesting one secondary hydroxyl oxidation took place at the non-reducing Xyl*p* moiety together with the oxidation at reducing end C-1. The Y_1_ ion shown in Figure 6E increased by 1 Da compared to the compound shown in Figure 6D, indicating that AmPDH1 can even generate triple oxidized X_2_ with a ketone group at the non-reducing Xyl*p* residue, a ketone at the reducing Xyl*p* residue, and a reducing end carboxylic acid.

Following the same procedure as described above for X_2_, the oxidized positions for other linear xylooligosaccharides were determined (Figure 5; see example MS^n^ spectra in Figures S16, S17). Oxidation by both AbPDH1 and AmPDH1 was clearly restricted to the reducing and non-reducing Xyl*p* residues. Consistent with quantification using ELSD (section *Separate Quantification of Xylooligosaccharides Oxidized at Secondary Hydroxyl Positions and the Reducing End C-1*), the MS fragmentation confirmed that linear xylooligosaccharides were mostly multi-oxidized by both PDHs, with the exception of X_4_ oxidation by AmPDH1 which was dominated by single oxidation. The MS fragmentation further revealed that the oxidation of X_4_ by AmPDH1 was localized to the reducing end secondary hydroxyls or reducing end C-1. Surprising, whereas single oxidation of X_4_ by both AbPDH1 and AmPDH1 was restricted to the reducing Xyl*p*, single oxidation of X_3_ by both PDHs was mainly at the non-reducing end Xyl*p* secondary hydroxyls (Figure 5). Double oxidation by PDHs included products oxidized at (1) secondary hydroxyls of both the reducing and non-reducing Xyl*p*, or (2) the reducing C-1 position and a secondary hydroxyl at the non-reducing end. Increasing the length of the xylooligosaccharide to X_3_ and X_4_ did not lead to detectable oxidation of internal Xyl*p* substituents. Interestingly, in addition to the regioselectivity of single and double oxidations, a product of *m/z* 299 was detected in the AmPDH1 oxidized X_2_ after reduction, representing products that were triple oxidized. In this case, it is conceivable that the reducing Xyl*p* is oxidized at both the reducing end C-1, and a secondary hydroxyl. This was the only detected triple oxidized product.

The Ara*f* substituted substrates were mainly single oxidized or non-oxidized after 24 h treatment. A^3^X containing α-(1 → 3)-linked Ara*f* substitution at the non-reducing end Xyl*p* residue, was single oxidized by both PDHs at the reducing end Xyl*p* resulting in a ketone group or C-1 oxidized carboxylic acid. A^2^XX with α-(1 → 2)-linked Ara*f* substitution was similarly oxidized at the secondary hydroxyl or C-1 of the reducing end Xyl*p* by both PDHs. Interestingly, in some A^2^XX products, a second ketone group was detected at the non-reducing end Xyl*p*, which carries the Ara*f* substituent. Moreover, AmPDH1 showed unique ability to form products with a single ketone group at the non-reducing end Xyl*p* of A^2^XX. No clear oxidation of Ara*f* was detected in any of the samples.

## Discussion

AbPDH1 was first isolated over 20 years ago (Volc et al., 1997), and was followed nearly 10 years later by the isolation of AmPDH1 (Sedmera et al., 2006). Since then, AbPDH1 and AmPDH1 have been biochemically characterized using a broad collection of monosaccharides, heteroglycosides, and selected oligosaccharides, revealing their potential for single and double oxidation of many plant-derived carbohydrates, and motivating their application in biofuel cells and organic syntheses (Giffhorn et al., 2000; Peterbauer and Volc, 2010). Herein, AbPDH1 and AmPDH1 were directly compared in terms of potential to oxidize diverse xylooligosaccharides, both linear and branched, acid and neutral. In particular, we investigated the impact of xylooligosaccharide length and substitution on the extent and position of oxidation by each enzyme. In this way, we evaluated the potential of AbPDH1 and AmPDH1 to serve as catalysts in the synthesis of telechelic building blocks from xylan fragments common to wood and agricultural residues.

Consistent with reported AmPDH1 activity toward X_2_ (Sygmund et al., 2008) and glucose-containing oligosaccharides (Volc et al., 1997; Tasca et al., 2007; Peterbauer and Volc, 2010; Rafighi et al., 2018) both AbPDH1 and AmPDH1 produced herein oxidized X_2_ as well as X_3_ and X_4_. As observed for PDH activity toward cellooligosaccharides and maltooligosaccharides (Kujawa et al., 2007; Sygmund et al., 2008; Peterbauer and Volc, 2010; Graf et al., 2017), the specific activity (U/mg) of both AbPDH1 and AmPDH1, as well as the rate of the substrate depletion detected with HPAEC-PAD, decreased with increasing degree of polymerization, where the impact of substrate length on activity was greater for AmPDH1 than for AbPDH1. Notably, AmPDH1 depleted X_2_ faster than AbPDH1, which is in line with the previous studies, where AmPDH1 shows higher catalytic turnover toward disaccharides, cellobiose, maltose and lactose than AmPDH1 (Sygmund et al., 2008; Gonaus et al., 2016). However, the reverse pattern was observed for longer linear oligosaccharides; AbPDH1 activity toward substituted xylooligosaccharides was also substantially higher than that measured for AmPDH1. In both cases, however, activities toward A^3^X were higher than X_3_, and activities toward neutral substrates were higher than for the acidic xylooligosaccharides U^4m2^XX and XU^4m2^XX. The structural basis for these differences in AbPDH1 and AmPDH1 activity toward oligosaccharides awaits a solved structure for AbPDH1 and of enzyme-substrate complexes for this enzyme family. Still, as found in Gonaus et al. (2016), a homology model of AbPDH1 based on the solved structure of AmPDH1 revealed two amino acid deletions in the loop 1 region, differences in the loop 3 region, and substitution of Phe508 in AmPDH1 to the smaller Val505 in AbPDH1. These differences vary the amino acid content in loop regions at the entrance of the substrate binding pocket, and could impact accessibility of the active site (Gonaus et al., 2016).

When considering the synthesis of telechelic molecules from xylooligosaccharides, it is especially important to evaluate the extent and positions of oxidation in each reaction product. To facilitate this analysis, we established an alternative method for the identification and quantification of oxidized carbohydrates, which utilizes NaBD_4_ reduction and is solely based on MS and UPLC systems. This method enables fast quantification of formed carbonyl groups as the results are readily interpreted by counting the Dalton difference. In addition, the analysis can be accomplished with reaction products in microgram levels, allowing it to be an attractive technique for characterizing carbohydrate oxidoreductases where enzyme or substrate quantities are limiting. For example, even though specific activity values could not be obtained using the acidic xylooligosaccharides U^4m2^XX and XU^4m2^XX, the near-complete oxidation of both substrates by AbPDH1 after 24 h was confirmed using this method. Given the higher activity of both AbPDH1 and AmPDH1 on neutral xylooligosaccharides, corresponding product profiles were quantified in more detail. Consistent with activity values, AbPDH1 reached a higher level of double oxidized end products from X_4_ and A^2^XX compared to AmPDH1. An exception was for X_2_, where treatment with AmPDH1 led even to a triple-oxidized product. The ESI-MS^n^ analyses confirmed that oxidations were targeted to the reducing and non-reducing ends of all tested xylooligosaccharide substrates. Interestingly, yet unexplained, selectivity was noticed by both enzymes. For example, a single keto group was formed at the non-reducing Xyl*p* residue of X_3_, whereas in X_4_ a single keto group was found solely at the reducing end Xyl*p*. Also, AmPDH1 produced more C-1 oxidized acidic products from X_3_ and X_4_ than AbPDH1. As previously reported for activity toward cellobiose, maltose and lactose (Volc et al., 2004; Sygmund et al., 2012; Gonaus et al., 2016), AbPDH1 and AmPDH1 oxidized the anomeric carbon together with secondary hydroxyls of xylooligosaccharides.

Although cross-ring fragmentation was observed in some oxidation products (e.g., with C-1 oxidation), we were not able to identify which secondary hydroxyl was targeted by each enzyme due to proton transfer during the fragmentation (Domon and Costello, 1988). Nevertheless, as reported for glucose-containing oligosaccharides (Sedmera et al., 2006; Peterbauer and Volc, 2010; Rafighi et al., 2018), the context of the secondary hydroxyls at C-2 and C-3 positions clearly impacted AbPDH1 and AmPDH1 activity toward xylooligosaccharides. Most notably, the alpha-(1 → 3)-linked Ara*f* in A^3^X and XA^3^XX shifted AbPDH1 and AmPDH1 activity toward the reducing end of both substrates. By contrast, both the reducing and non-reducing ends of A^2^XX were oxidized.

In summary, with the newly developed method to identify and quantify oxidized carbohydrates, we successfully determined the degree of oxidation and analyzed the regioselectivity of AbPDH1 and AmPDH1 toward xylooligosaccharides present in wood and agricultural fiber. The widened carbohydrate profile of AbPDH1 and AmPDH1 provides further support for their proposed biological function in evading plant defense mechanisms through reducing plant-derived quinones, or promoting lignin depolymerization through reducing lignin-derived radicals generated by lignin-active peroxidases and laccases (Peterbauer and Volc, 2010; Sützl et al., 2018; Herzog et al., 2019). Confirming herein that oxidative activity is restricted to the ends of xylooligosaccharides, and that both linear and substituted xylooligosaccharides are accepted, also opens new applications of PDHs that transform underused xylan streams into telechelic molecules primed for polymerization.

## Data Availability Statement

The datasets generated for this study are available on request to the corresponding author.

## Author Contributions

JK, HZ, AK, MT, and EM contributed conception and design of the study. JK, HZ, and AK performed the experiments. JK, HZ, S-LC, and AK analyzed the data. MT and EM contributed reagents, materials, and analysis tools. JK, HZ, MT, and EM wrote the paper. All authors read and approved the submitted version.

### Conflict of Interest

The authors declare that the research was conducted in the absence of any commercial or financial relationships that could be construed as a potential conflict of interest.
