# Supplementary material for: Quantitative Comparison of Pyranose Dehydrogenase Action on Diverse Xylooligosaccharides
Source: Front Chem. 2020 Jan 28;8:11. doi: 10.3389/fchem.2020.00011 (PMC6997461; doi:10.3389/fchem.2020.00011)
Supplement: Supplementary file 1 [file Data_Sheet_1.PDF]

Supplementary Material

Content list

Table S1. Optimal pH of glucose oxidation reactions with 1,4-benzoquinone (BQ) and ferrocenium ion (Fc) as e-acceptors.

Table S2. The possible chemical structures of reaction products of xylotriose oxidized by AbPDH1 for 24 hours.

Figure S1. Negative-mode ESI-MS spectrum of xylotriose (X_3).

Figure S2. SDS-PAGE of purified native and deglycosylated AbPDH1 and AmPDH1.

Figure S3. Normalized specific activities of AbPDH1 and AmPDH1 in different reaction conditions.

Figure S4. Effect of laccase and buffer conditions in PDH oxidation of xylose.

Figure S5. Analysis of 3^2-α-L-arabinofuranosyl-xylobiose (AX) on HPAEC-PAD.

Figure S6. Analysis of 2^3-α-L-arabinofuranosyl-xylotriose (AXX) on HPAEC-PAD.

Figure S7. Analysis of 3^3-α-L-arabinofuranosyl-xylotetraose (XAXX) on HPAEC-PAD.

Figure S8. Substrate depletion of the 4 mM 2^3-(4-*O*-methyl-α-D-glucuronyl)-xylotriose ($U^{4m2}XX$) and 2^3-(4-*O*-methyl-α-D-glucuronyl)-xylotetraose ($XU^{4m2}XX$).

Figure S9. Negative ion MS spectra of linear xylooligosaccharidees.

Figure S10. Negative ion MS spectra of Ara*f* substituted xylooligosaccharides.

Figure S11. Negative ion MS spectra of 2^3-(4-*O*-methyl-α-D-glucuronyl)-xylotriose ($U^{4m2}XX$) and 2^3-(4-*O*-methyl-α-D-glucuronyl)-xylotetraose ($XU^{4m2}XX$).

Figure S12. Negative MS spectra of $NaBD_4$ reduced linear xylooligosaccharides.

Figure S13. Negative MS spectra of $NaBD_4$ reduced Ara*f* substituted xylooligosaccharides.

Figure S14. UPLC chromatogram of linear xylooligosaccharides.

Figure S15. UPLC chromatogram of Ara*f* substituted xylooligosaccharides.

Figure S16. Extract of negative ion MS^n spectra of xylotriose (X_3) — Part one.

Figure S17. Extract of negative ion MS^n spectra of xylotriose (X_3) — Part Two.

Table S1. Optimal pH of glucose oxidation reactions with 1,4-benzoquinone (BQ) and ferrocenium ion (Fc) as e-acceptors.

Enzyme	Production host[1]	Substrate	Substrate (mM)	e-acceptor	e-acceptor (mM)	Studied pH range	pH optimum (1st)	pH optimum (2nd)	Buffer	Buffer pH range	Buffer (mM)	Temperature	Buffer effect[2]	Reference
AbPDH1	n	glucose	25	BQ	2.3	pH 2.5-9.5	9		Na-phosphate	pH 6-9.5	100	25		Volc et al. 1997
AbPDH1	n	glucose	25	BQ	2.3	pH 2.5-9.5		4	Na-acetate	pH 3-6.5	100	25	Na-malonate, pH 5	Volc et al. 1997
AbPDH1	*r_pp*	*glucose*	*25*	*BQ*	*5*	*pH 3.5-7*	*7*		*Na-phosphate*	*pH 6-7*	*50*	*30*		*This manuscript*
AbPDH1	*r_pp*	*glucose*	*25*	*BQ*	*5*	*pH 3.5-7*		*5.5*	*Na-acetate*	*pH 3.5-5.5*	*50*	*30*	*NH4-acetate, pH 4.5*	*This manuscript*
AcPDH1	r_pp	glucose	25	BQ	2	pH 2.5-8.5	7		K-phosphate	pH 6-8	100	30		Staudigl et al. 2013
AcPDH1	r_pp	glucose	25	BQ	2	pH 2.5-8.5		5.5	Na-citrate	pH 2.5-6	100	30		Staudigl et al. 2013
AmPDH1	n	glucose	25	BQ	2.3	pH 1-6.5	2		Na-citrate	pH 1-3	50	25		Sygmund et al. 2008
AmPDH1	r_pp	glucose	25	BQ	Na	pH 2-8	2		Na-citrate	pH 2.5-6	100	30		Sygmund et al. 2012
AmPDH1	*r_pp*	*glucose*	*25*	*BQ*	*5*	*pH 3.5-7*	*4*		*Ammonium-acetate*	*pH 3.5-5.5*	*50*	*30*	*Na-acetate, pH 4.5*	*This manuscript*
AmPDH1	r_an	glucose	25	BQ	Na	pH 2-10	9		borate	pH 8-10	100	30		Pisanelli et al. 2010
AmPDH1	r_pp	glucose	25	BQ	Na	pH 3-9	9		Britton–Robinson	pH 3-9	40	30		Graf et al. 2017
AmPDH2	r_pp	glucose	25	BQ	Na	pH 3-9	8.5		Britton–Robinson	pH 3-9	40	30		Graf et al. 2017
AmPDH3	r_pp	glucose	25	BQ	Na	pH 3-9	9		Britton–Robinson	pH 3-9	40	30		Graf et al. 2017
AxPDH1	n	glucose	25	BQ	Na	pH 2-8	2.5		Na-citrate	pH 2-6	100	30		Kujawa et al. 2007
AxPDH1	n	glucose	25	BQ	Na	pH 2-8		8	phosphate	pH 6-8	100	30		Kujawa et al. 2007
AxPDH1	r_pp	glucose	25	BQ	2	pH 2.5-9	5.5		Na-citrate	pH 2-6	100	30		Staudigl et al. 2013
AxPDH1	r_pp	glucose	25	BQ	2	pH 2.5-9		8	borate	pH 8-9	100	30		Staudigl et al. 2013
AbPDH1	r_pp	glucose	25	Fc	0.2	pH 3-8.6	8.6		Na-phosphate	pH 6-8.6	50	30		Gonaus et al. 2016
AcPDH1	r_pp	glucose	25	Fc	0.2	pH 2.5-10	8.5		borate	pH 8-10	100	30		Staudigl et al. 2013
AmPDH1	n	glucose	25	Fc	0.2	pH 2.5-9	9		bisTris–propane·HCl	pH 6.5-9	50	30		Sygmund et al. 2008
AmPDH1	r_an	glucose	25	Fc	Na	pH 2-10	10		borate	pH 8-10	100	30		Pisanelli et al. 2010
AmPDH1	r_pp	glucose	25	Fc	0.4	pH 2-9	9		borate	pH 8-9	100	30		Sygmund et al. 2012
AmPDH1	r_pp	glucose	25	Fc	0.2	pH 2.5-10	9		borate	pH 8-10	100	30		Krondorfer et al. 2014
AmPDH1	r_pp	glucose	25	Fc	0.2	pH 3-8.6	8.6		Na-phosphate	pH 6-8.6	50	30		Gonaus et al. 2016
AmPDH1	r_pp	glucose	25	Fc	0.2	pH 3-9	9		Britton–Robinson	pH 3-9	40	30		Graf et al. 2017
AmPDH2	r_pp	glucose	25	Fc	0.2	pH 3-9	9		Britton–Robinson	pH 3-9	40	30		Graf et al. 2017
AmPDH3	r_pp	glucose	25	Fc	0.2	pH 3-9	9		Britton–Robinson	pH 3-9	40	30		Graf et al. 2017
AxPDH1	n	glucose	25	Fc	0.4	pH 2-10	8.5		borate	pH 8-10	100	30		Kujawa et al. 2007
AxPDH1	r_pp	glucose	25	Fc	0.2	pH 2.5-10	9		borate	pH 8-10	100	30		Staudigl et al. 2013

[1]n: native; r_pp: recombinant produced in *Pichia pastoris*; r_an: recombinant produced in *Aspergillus niger*. [2]Detected optimum pH depends on the selected and studied buffering agent in same pH range. Studied second buffer and its optimum pH is reported.

Table S2. The possible chemical structures of reaction products of xylotriose oxidized by AbPDH1 for 24 hours.

Code	Chemical sturcture	[M+Cl]$^-$ m/z	[M-H]$^-$ m/z
A	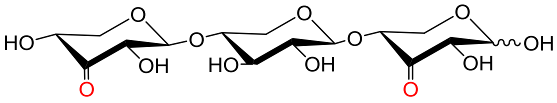	**445**	409
B+H_2O	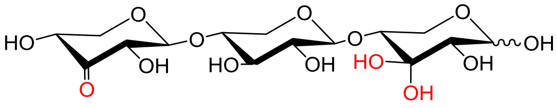	**463**	427
A+2H_2O	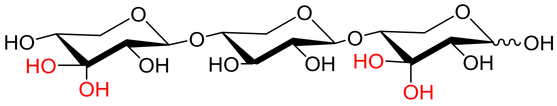	**481**	445
B	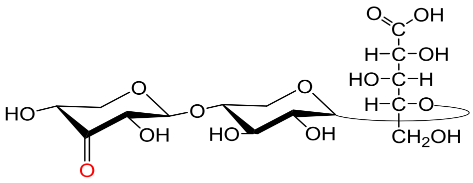	463	**427**
B+H_2O	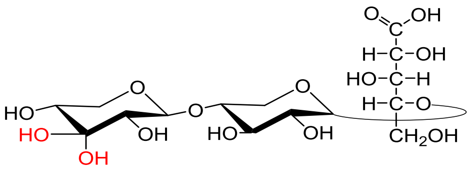	481	**445**

A

Example calculation of oxidized xylotriose, using the system of equations developed herein and the mass spectrum shown below (C).

The monoisotopic mass of X_3 has a *m/z* of 452 (B), and the naturally present isotopic chloride, carbon, hydrogen and oxygen generate *m/z* of 453 and 454. Since the secondary hydroxyl oxidations were labeled with deuterium, the *m/z* 453 consists of single oxidized X_3 and the isotopic non-oxidized X_3 while the *m/z* 454 consists of double oxidized X_3, the isotopic non-oxidized X_3, and the isotopic mono-oxidized X_3.

Applying Equations (1-4), the degree of oxidation can then be calculated for oxidations at secondary hydroxyls as follows:

$$\begin{aligned}\text{Abundance of isotopic m/z 452 in (B)} &= \text{abundance of non-oxidized } X_3 \\ &+ \text{abundance of single oxidized } X_3 + \text{abundance of double oxidized } X_3 \\ &= a + (b - a \times r1 \div 100) \\ &+ \{c - a \times r2 \div 100 - (b - a \times r1 \div 100) \times r1 \div 100\} \qquad (1)\end{aligned}$$

where a, b, and c are the abundance of m/z 452, m/z 453, and m/z 454, respectively, after the AbPDH1 oxidation; and r1 and r2 are the relative abundance of naturally present isotopic X_3.

$$\text{Non-oxidation\%} = 100\% - \text{single oxidation\%} - \text{double oxidation\%} \qquad (2)$$

$$\text{Single oxidation\%} = \frac{\text{Abundance of single oxidized } X_3}{\text{Abundance of isotopic } m/z \text{ 452 in control } X_3 \text{ (B)}} \times 100\% \qquad (3)$$

$$\text{Double oxidation\%} = \frac{\text{Abundance of double oxidized } X_3}{\text{Abundance of isotopic } m/z \text{ 452 in control } X_3 \text{ (B)}} \times 100\% \qquad (4)$$

When X_3 is oxidized at the reducing end C-1, the reaction product containing carboxyl acid show in anionic, deprotonated form with a *m/z* of 429. The theoretical isotopic distribution of C-1 oxidized X_3 was generated by Masslynx V4.1 as shown below (D). When additional oxidations take place at the secondary hydroxyls, the deuterium label will generate peaks with the correspondind increase in Daltons. As can be seen below in (E), the C-1 oxidation is mostly accompanied by one or two secondary hydroxyl oxidations. Therefore, by applying Equations (5-8), the extent of oxidation with at least one oxidation at reducing end C-1 can be calculated as follows:

$$\begin{aligned}\text{Abundance of isotopic } m/z \text{ 429 in (D)} &= \text{abundance of C-1 oxidized } X_3 \\ &+ \text{abundance of double oxidized } X_3 + \text{abundance of triple oxidized } X_3 \\ &= a' + (b' - a' \times r1' \div 100) \\ &+ \{c' - a' \times r2' \div 100 - (b' - a' \times r1' \div 100) \times r1' \div 100\} \qquad (5)\end{aligned}$$

where a’, b’, and c’ are the abundance of m/z 429, m/z 430, and m/z 431, respectively, after the AbPDH1 oxidation; and r1’ and r2’ are the relative abundance of theoretically generated isotopic X_3 with one oxidation at reducing end C-1. The double oxidation consists of one reducing end C-

1 oxidation and one secondary hydroxyl oxidation. The triple oxidation consists of one reducing end C-1 oxidation and two secondary hydroxyl oxidation.

$$\text{Reducing end C-1 oxidation\%= 100\%} - \text{double oxidation\%} - \text{triple oxidation\%} \quad (6)$$

$$\text{Double oxidation\%=}\frac{\text{Abundance of double oxidized } X_3}{\text{Abundance of isotopic } m/z \text{ 429 in theoretical (D)}} \times 100\% \quad (7)$$

$$\text{Triple oxidation\%=}\frac{\text{Abundance of triple oxidized } X_3}{\text{Abundance of isotopic } m/z \text{ 429 in theoretical (D)}} \times 100\% \quad (8)$$

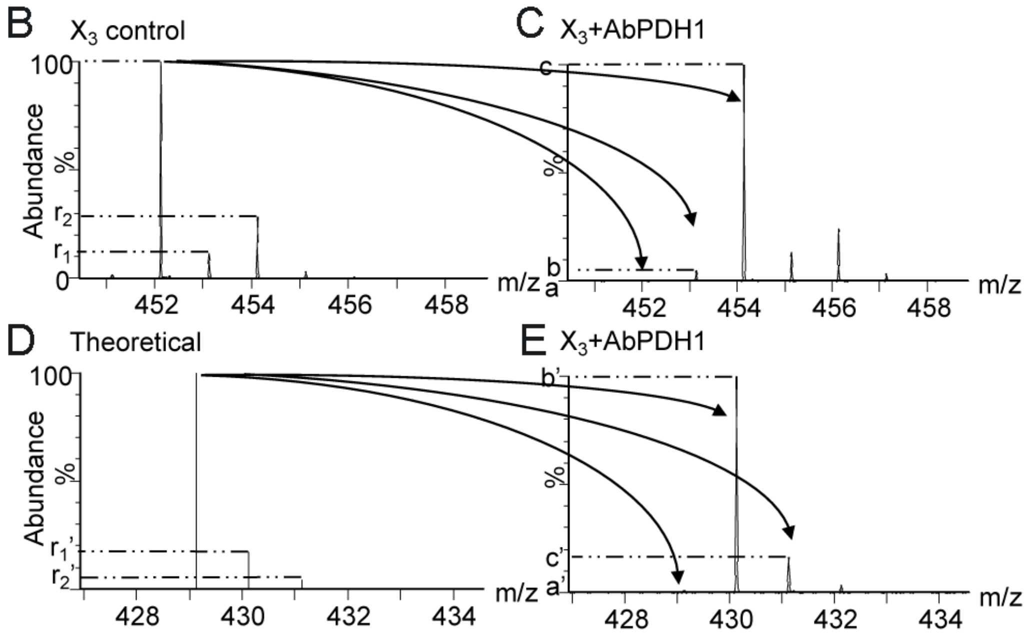

Figure S1. Quantitative interpretation of mass spectra. (A) Example calculations, negative-mode ESI-MS spectrum of (B) reduced xylotriose (X_3) in control reaction, Cl^- adduct, (C) products of X_3 oxidized by AbPDH1 at secondary hydroxyls after reduction, Cl^- adduct, (D) the theoretical isotopic distribution of reduced X_3 with one oxidation at reducing end C-1, (E) the products of X_3 oxidized by AbPDH1 with at least one oxidation at reducing end C-1.

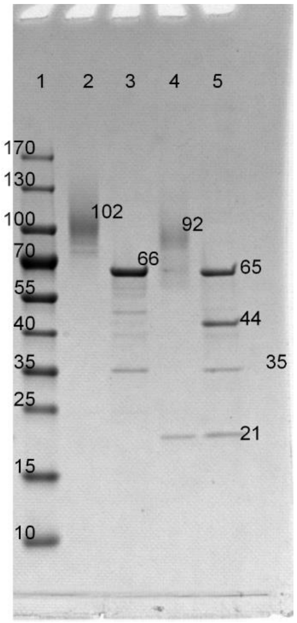

Figure S2. SDS-PAGE of purified and deglycosylated AmPDH1 and AbPDH1. Lane 1, molecular weight standard; lane 2, AmPDH1; lane 3, deglycosylated AmPDH1; lane 4, AbPDH1; lane 5, deglycosylated AbPDH1. AbPDH1 was fragmented during the SDS-PAGE run (confirmed by native page and MS, data not shown). N-deglycosidase (PNGase F) is shown by the 35 kDa band in deglycosylated samples. The purity, size and glycosylation of the produced and purified PDHs were estimated with SDS-PAGE. SDS-PAGE was conducted based on manufacturer's protocol using 4-20% gradient protein gels (Mini-protean TGX, Bio-Rad Laboratories, USA) and PageRulerTM Prestained protein ladder (Thermo Fisher, USA) was used as a standard. Proteins were stained using commercial Coomassie blue stain (Thermo Scientific, USA). Stained gels were imaged and molecular weight was determined using Image Lab (Bio-Rad Laboratories, USA). Produced proteins were deglycosylated with peptide:N-glycosidase F (PNGase F, New England Biolabs, USA). All deglycosylations were done in denaturing conditions according to manufacturer's instructions. Purification of the PDHs were conducted as follows. After induction, cells were collected by centrifugation (15 000 g, 20 min, 4°C) and supernatants were filtered through a 2-fold Miracloth (Merck, Germany) and 0.45 μm PES filter (Merck, Germany), and then concentrated using a Vivaflow 200 10 kDa cut off crossflow filter (Sartorius, Germany). The filtrate was adjusted to pH 7.4 and then loaded onto 7 ml Ni-NTA matrix (Qiagen, Germany) packed in a XK-16/10 column (GE Life Sciences, Germany). The column matrix was equilibrated in binding buffer (50 mM sodium phosphate buffer with 500 mM sodium chloride and 20 mM imidazole, pH 7.4). After sample loading, the column matrix was washed with 5-7 column volumes of binding buffer and bound proteins were eluted in 5 column volumes using a linear gradient (0-100%) of elution buffer (50 mM sodium phosphate buffer with 500 mM sodium chloride and 500 mM imidazole, pH 7.4). AbPDH1 was further purified using a Hitrap Capto Q (GE healthcare, USA) anion exchange column and 20 mM triethylamine (pH 7.5) as the binding buffer; the protein was eluted with increasing NaCl concentration stepwise from 0 to 0.5 M. Finally, AbPDH1 was polished using a HiLoad 16/600 Superdex 200 pg column (GE healthcare, USA) in 10 mM HEPES pH 7.5 with 0.15 M NaCl. Protein purity was estimated at each step by SDS-PAGE. Purified fractions of AbPDH1 and AmPDH1 were pooled, and exchanged to 10 mM HEPES buffer (pH 7.5) using 30 kDA cut off Vivaspin 20 spin columns (Sartorius, Germany).

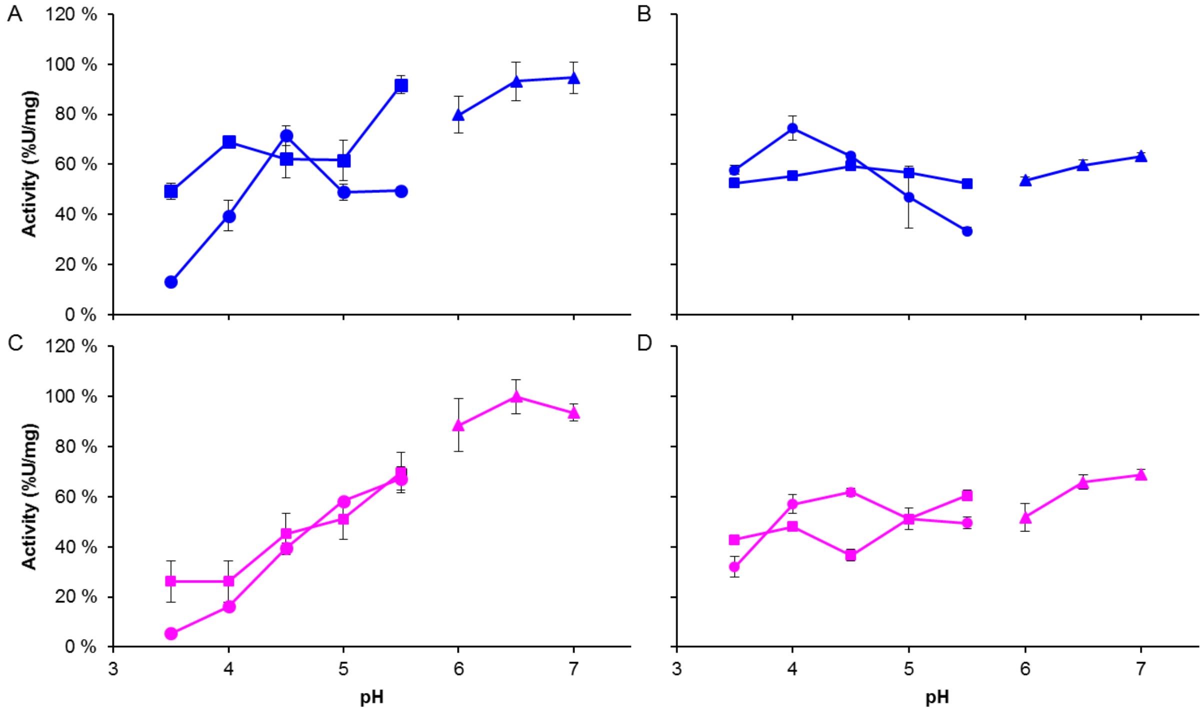

Figure S3. Normalized specific activities of AbPDH1 (A and C) and AmPDH1 (B and D) on 25 mM glucose (blue) and 25 mM xylose (pink). Reactions contained 50 mM of the following buffers: sodium acetate (square, pH 3.5 to 5.5), ammonium acetate (circle, pH 3.5 to 5.5) and sodium phosphate (triangle, pH 6 to 7) with 0.5 pH unit increase. Reactions were conducted based on the general activity method described in the main text with 5 mM 1,6-benzoquinone as the electron acceptor and results were normalized towards AbPDH1 activity on xylose at pH 6.5.

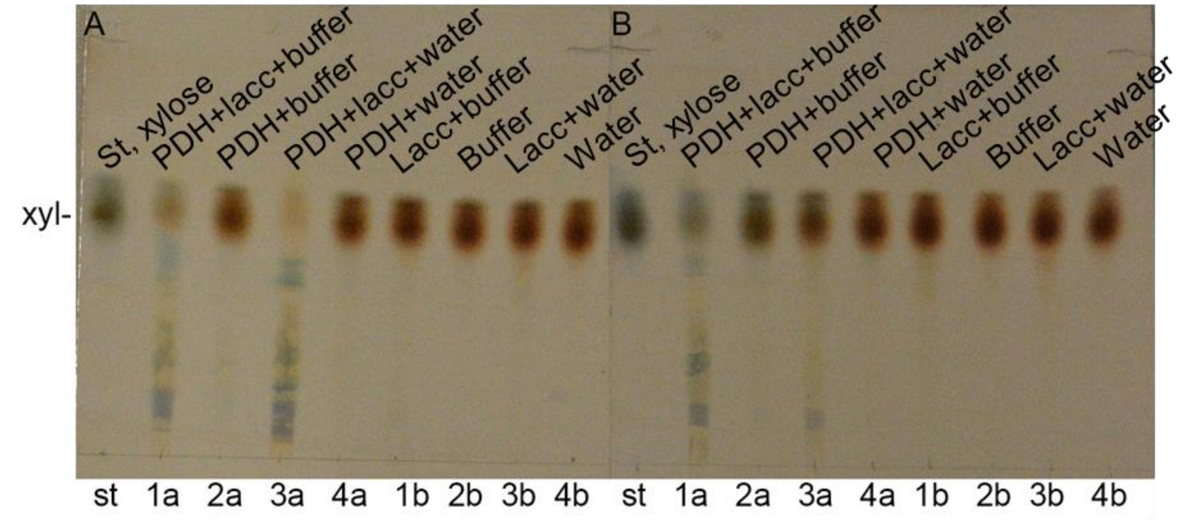

C

Enzyme	Buffer	Comparison pairs	Xylose depletion (%±st)
AbPDH1	Na-acetate, pH 5	1a to 1b	89±2.5
AbPDH1	No buffer	3a to 3b	31±13
AmPDH1	Na-citrate, pH 3	1a to 1b	85±11
AmPDH1	No buffer	3a to 3b	98±3.5

Figure S4. Effect of laccase and buffer conditions in PDH oxidation of xylose. Oxidation of 50 mM xylose with (A) 1 U of AbPDH1 and (B) 1 U of AmPDH1 with and without 1 U of laccase (Sigma-Aldrich, Germany) were conducted in 96-well plates in 250 µl volume at 30°C for 24 h and with 300 rpm mixing. AbPDH1 and AmPDH1 reactions were done in 10 mM Na-acetate buffer (pH 5.0) and in 10 mM Na-citrate buffer (pH 3.0), respectively, as well as and in RO-water. Electron acceptor (BQ) concentration was 5 mM. All the reactions and controls were performed in duplicate and one replicate of each reaction was analyzed by thin layer chromatography (TLC) and HPAEC-PAD. For TLC, 1 µl of reaction product was spotted on the silica plate (Silica Gel 60, Merck, USA), dried, and then resolved using 1-butonal-pyridine-boric acid (saturated in RO-water)-acetic acid at a volumetric ratio of 6:4:3:1 as the eluent. TLC plates were stained using the diphenylamine-aniline reagent, using a solution containing 2% (w/v) diphenylamine, 1.8% (v/v) aniline, and 8.9 % (v/v) phosphoric acid. (C) Xylose depletion in the reactions was determined with HPAEC-PAD (DionexTM-5000+ with DionexTM CarboPacTM PA1 IC column, Thermo Scientific, USA), analyzing duplicate reactions with laccase and without laccase. Before analysis, enzymes were removed from the reaction using a 10 kDa cut off Vivaspin 500, and the filtrate was diluted to 90-100 ppm carbohydrate concentration. Eluents were 100 mM NaOH and 100 mM NaOH with 1 M NaOAc. Depletion of the xylose peak in reactions containing both a PDH and laccase was compared to reactions containing laccase as the sole enzyme.

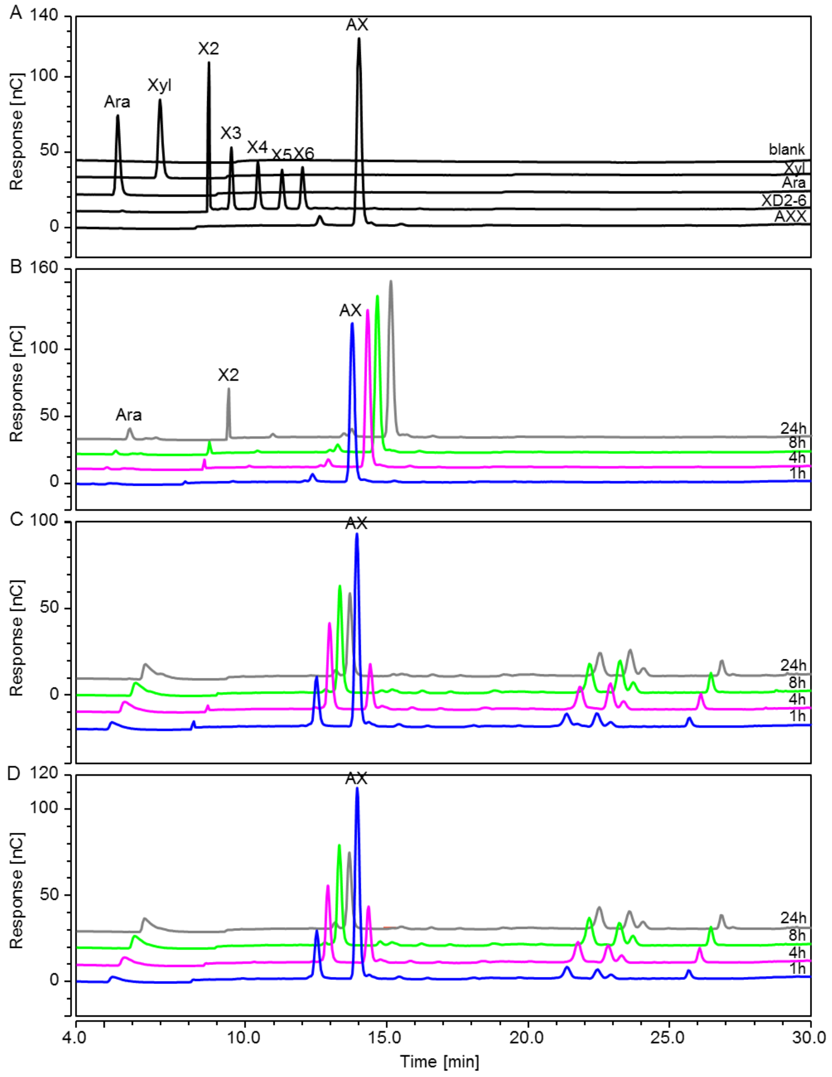

Figure S5. Analysis of 3^2-α-L-arabinofuranosyl-xylobiose (AX) by HPAEC-PAD. (A) Standards: AX, 3^2-α-L-arabinofuranosyl-xylobiose 100 ppm; XD2-6, standard mixture of xylobiose to xylohexaose, 25 ppm; Ara, arabinose, 25 ppm; Xyl, xylose, 25 ppm and blank; mQ-H_2O. 5 mM AX (B) control reaction with all the reaction components, ie. 1 mM BQ and 0.2 U/ml of laccase, and without PDH1, (C) reaction with 0.2 U/ml AbPDH1, and (D) reaction with 0.2 U/ml AmPDH1, time points 1 h (blue), 4 h (pink), 8 h (green), and 24 h (grey). Reactions were performed at 30°C for up to 48 h with shaking (500 rpm) in 10 mM ammonium-acetate buffer (pH 5.5). The laccase preparation had minor α-arabinofuranosidase activity releasing xylobiose (X2) and arabinose, and resulting in 7.5% loss of AX after 24 h. All the samples were adjusted to 100 ppm of carbohydrate, and the injected volume was 10 µl. Example chromatograms are shown, and for illustration purposes, have been separated from the adjacent by 7% signal and 2% time offsets.

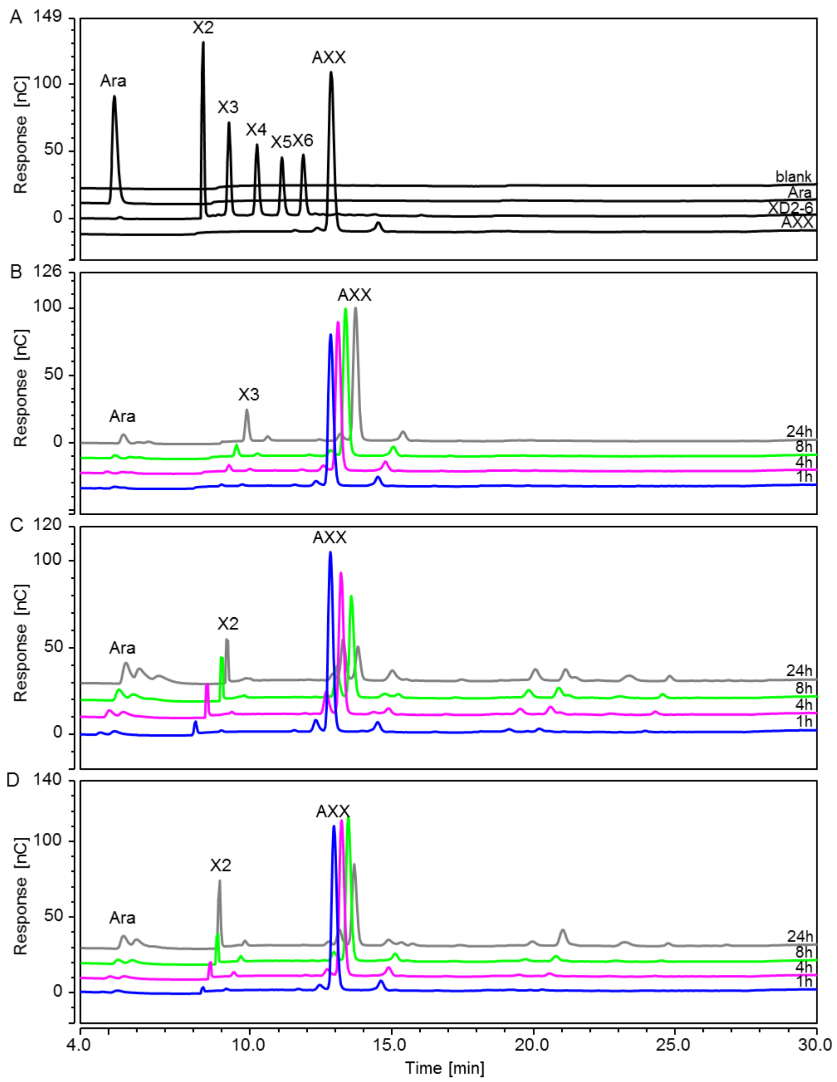

Figure S6. Analysis of 2^3-α-L-arabinofuranosyl-xylotriose (AXX) by HPAEC-PAD. (A) Standards: AXX, 2^3-α-L-arabinofuranosyl-xylotriose, 100 ppm; XD2-6, standard mixture of xylobiose to xylohexaose, 25 ppm; Ara, arabinose, 25 ppm; and blank; mQ-H2O. 5 mM A2XX (B) control reaction with all the reaction components, ie. 1 mM BQ and 0.2 U/ml of laccase, and without PDH1, (C) reaction with 0.2 U/ml AbPDH1, and (D) reaction with 0.2 U/ml AmPDH1, time points 1 h (blue), 4 h (pink), 8 h (green), and 24 h (grey). Reactions were performed at 30°C for up to 48 h with shaking (500 rpm) in 10 mM ammonium-acetate buffer (pH 5.5). The laccase preparation had minor α-arabinofuranosidase activity releasing of xylotriose (X3) and arabinose, and resulting in 20% loss of AXX after 24h. In addition, minor degradation of xylooligosaccharide backbone was detected with HPAEC-PAD, resulting in the formation of xylobiose (X2) and X3 peaks. However, mass spectrometric analysis of corresponding samples did not indicate presence of X2 after 24 h reaction, indicating that the xylooligosaccharide degradation occurred during the HPAEC-PAD run possibly through beta-elimination in the alkaline conditions. All the samples were adjusted to 100 ppm of carbohydrate, and the injected volume was 10 µl. Example chromatograms are shown, and for illustration purposes, have been separated from the adjacent by 7% signal and 2% time offsets.

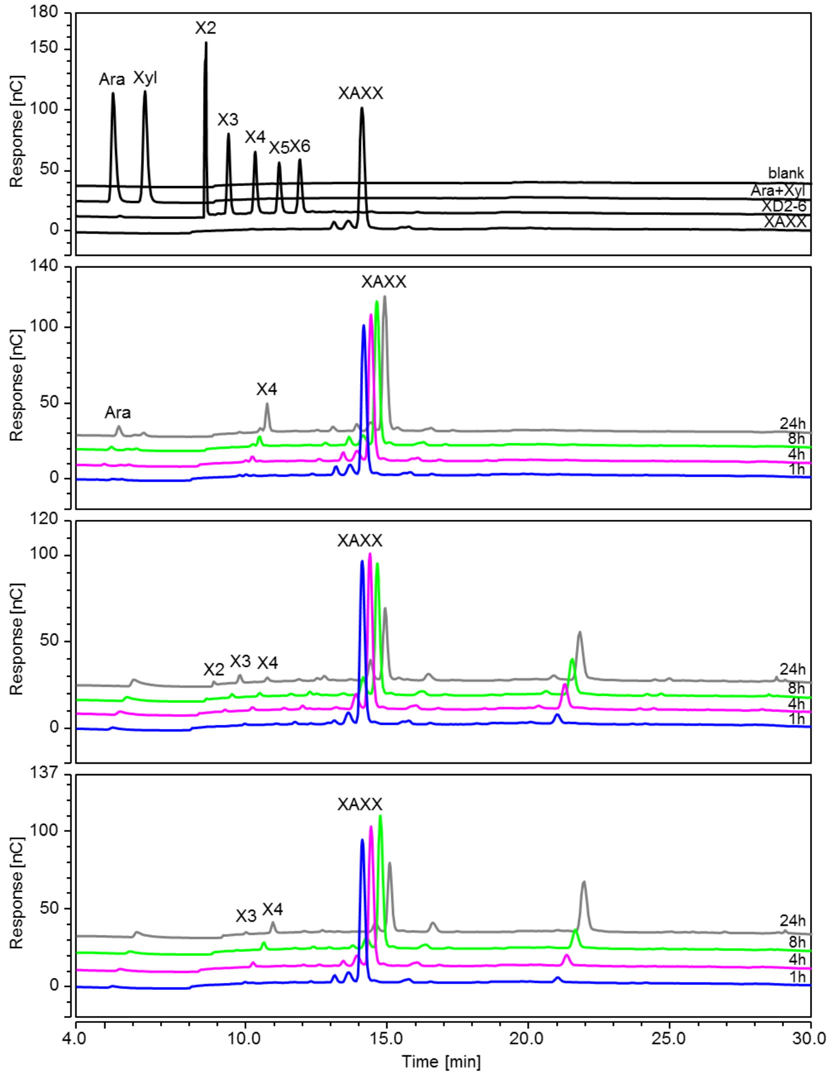

Figure S7. Analysis of 3^3-α-L-arabinofuranosyl-xylotetraose (XAXX) by HPAEC-PAD. (A) Standards: XAXX, 3^3-α-L-arabinofuranosyl-xylotetraose, 100 ppm; XD2-6, standard mixture of xylobiose to xylohexaose, 25 ppm; Ara, arabinose, 25 ppm; and blank; mQ-H2O. (B) 5 mM XAXX control reaction with all the reaction components, ie. 1 mM BQ and 0.2 U/ml of laccase, and without PDH1, (C) reaction with 0.2 U/ml AbPDH1, and (D) reaction with 0.2 U/ml AmPDH1, time points 1 h (blue), 4 h (pink), 8 h (green), and 24 h (grey). Reactions were performed at 30°C for up to 48 h with shaking (500 rpm) in 10 mM ammonium-acetate buffer (pH 5.5). The laccase preparation had minor α-arabinofuranosidase activity releasing of xylotetraose (X4) and arabinose, and resulting in 11.6% loss of XAXX after 24h. In addition, minor degradation of xylooligosaccharide backbone was detected with HPAEC-PAD, resulting in the formation of xylobiose (X2), xylotriose (X3) and xylotetraose (X4) peaks. However, mass spectrometric analysis of corresponding samples did not indicate presence of X2 and X3 after 24 h reaction, indicating that the xylooligosaccharide degradation occurred during the HPAEC-PAD run possibly through beta-elimination in the alkaline conditions. All the samples were adjusted to 100 ppm of carbohydrate, and the injected volume was 10 µl. Example chromatograms are shown, and for illustration purposes, have been separated from the adjacent by 7% signal and 2% time offsets.

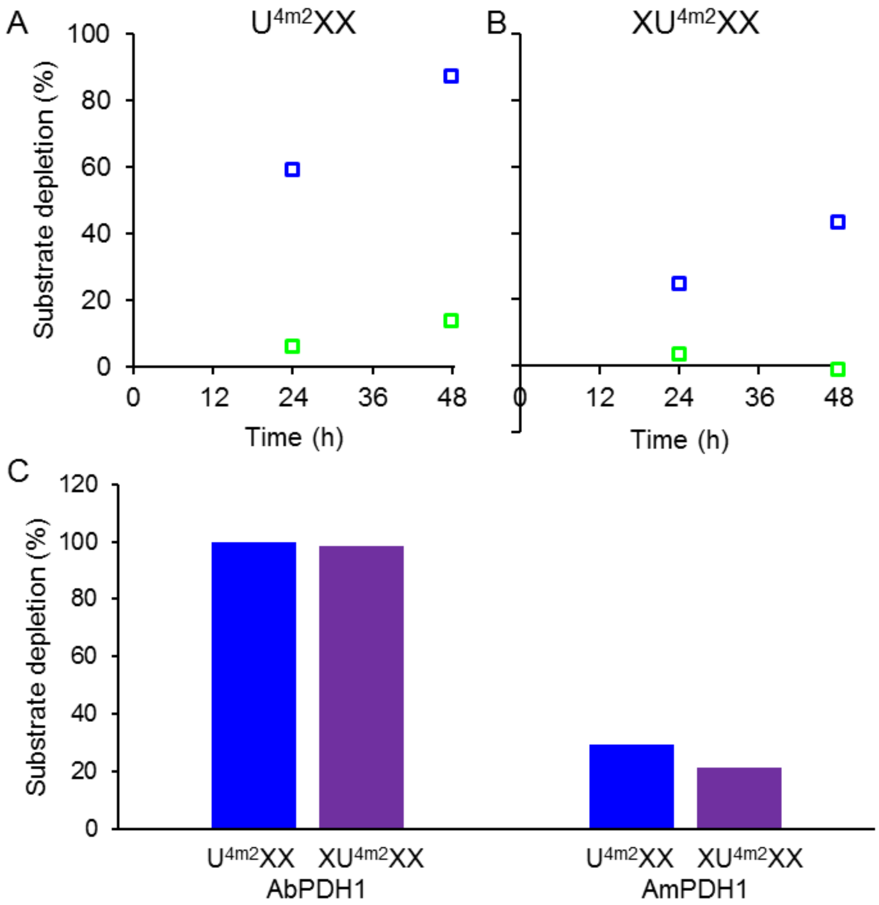

Figure S8. HPAEC analysis of AbPDH1 and AmPDH1 conversion of 4 mM 2^3-(4-*O*-methyl-α-D-glucuronyl)-xylotriose ($U^{4m2}XX$) and 2^3-(4-*O*-methyl-α-D-glucuronyl)-xylotetraose ($XU^{4m2}XX$). (A) Time course of single reactions (30 µl) containing 4 mM $U^{4m2}XX$ and 0.2 U/ml of AbPDH1 (blue) or 0.2 U/ml of AmPDH1 (green), (B) time course of single reactions (30 µl) containing 4 mM $XU^{4m2}XX$ and 0.2 U/ml of AbPDH1 (blue) or 0.2 U/ml of AmPDH1, (C) duplicate reactions (400 µl) showing % substrate depletion after 24 h, where reactions contained 2 U/ml of AbPDH1 and AmPDH1; the data range was less than 1.4%. All reactions contained 1 mM BQ and 0.2 U/ml laccase.

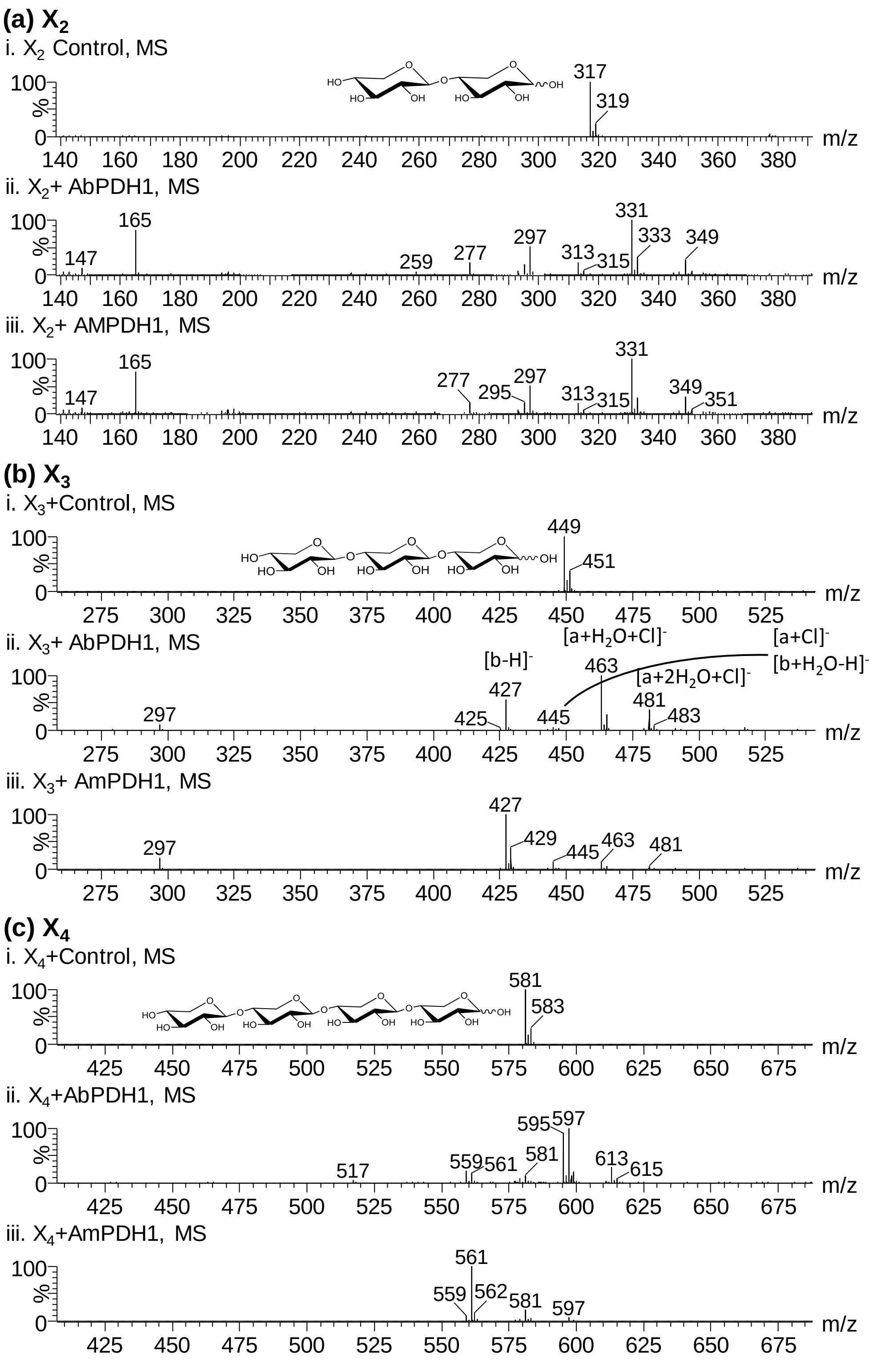

Figure S9. Negative ion MS spectra of (A) xylobiose (X_2), (B) xylotriose (X_3), and (C) xylotetraose (X_4). Control reactions, AbPDH1 oxidation, and AmPDH1 oxidation shown as i, ii, iii, respectively. Non-oxidized X_2, X_3, X_4 ionized as chloride adducts with *m/z* of 317, 449, 581 respectively.

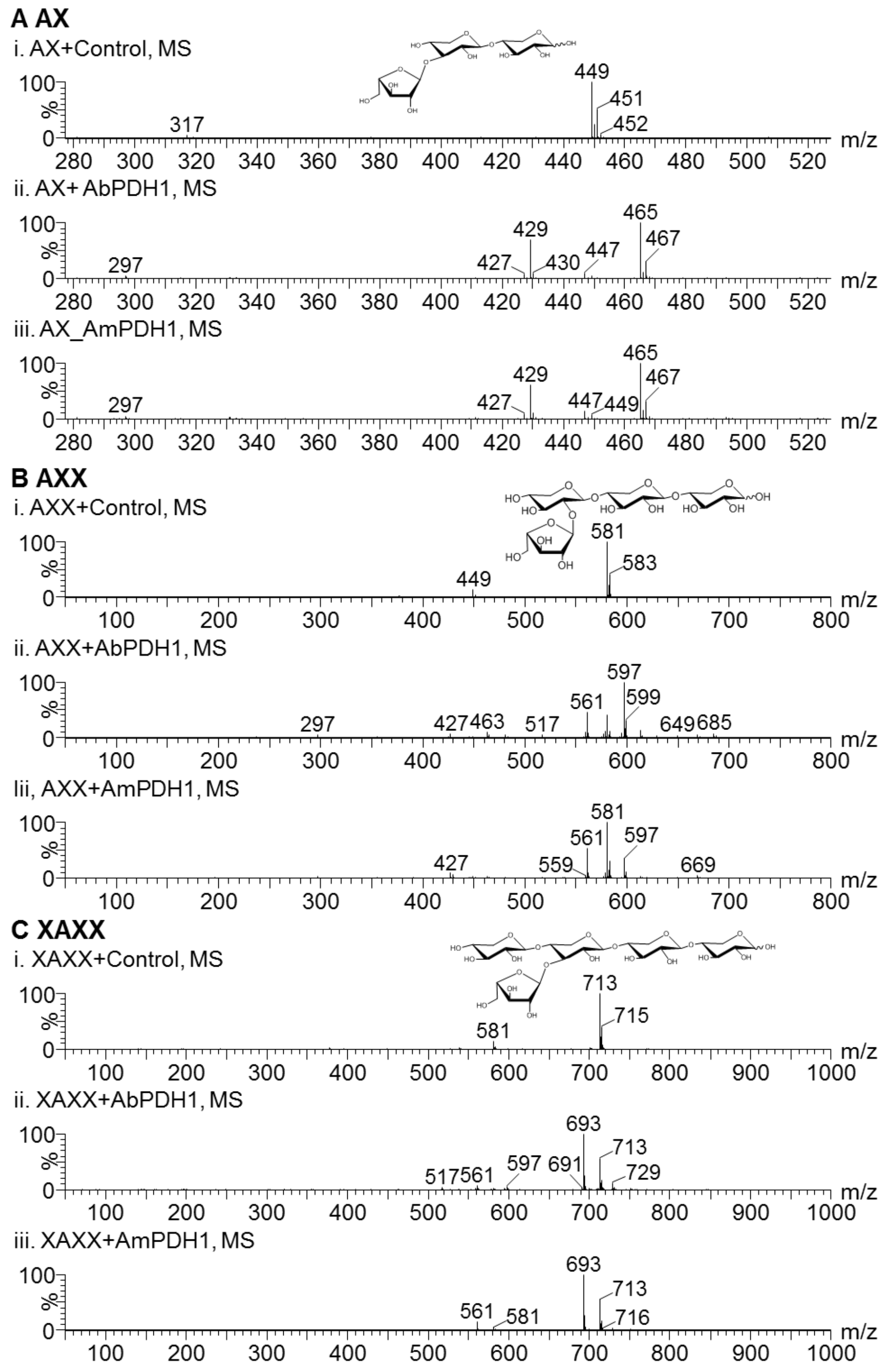

Figure S10. Negative ion MS spectra of (A) 3^2-α-L-arabinofuranosyl-xylobiose (AX), (B) 2^3-α-L-arabinofuranosyl-xylotriose (AXX), (C) 3^3-α-L-arabinofuranosyl-xylotetraose (XAXX); control reactions, AbPDH1 oxidation, and AmPDH1 oxidation were shown as i, ii, iii, respectively. Non-oxidized AX, AXX, XAXX ionized as chloride adducts with *m/z* of 449, 581, 713 respectively.

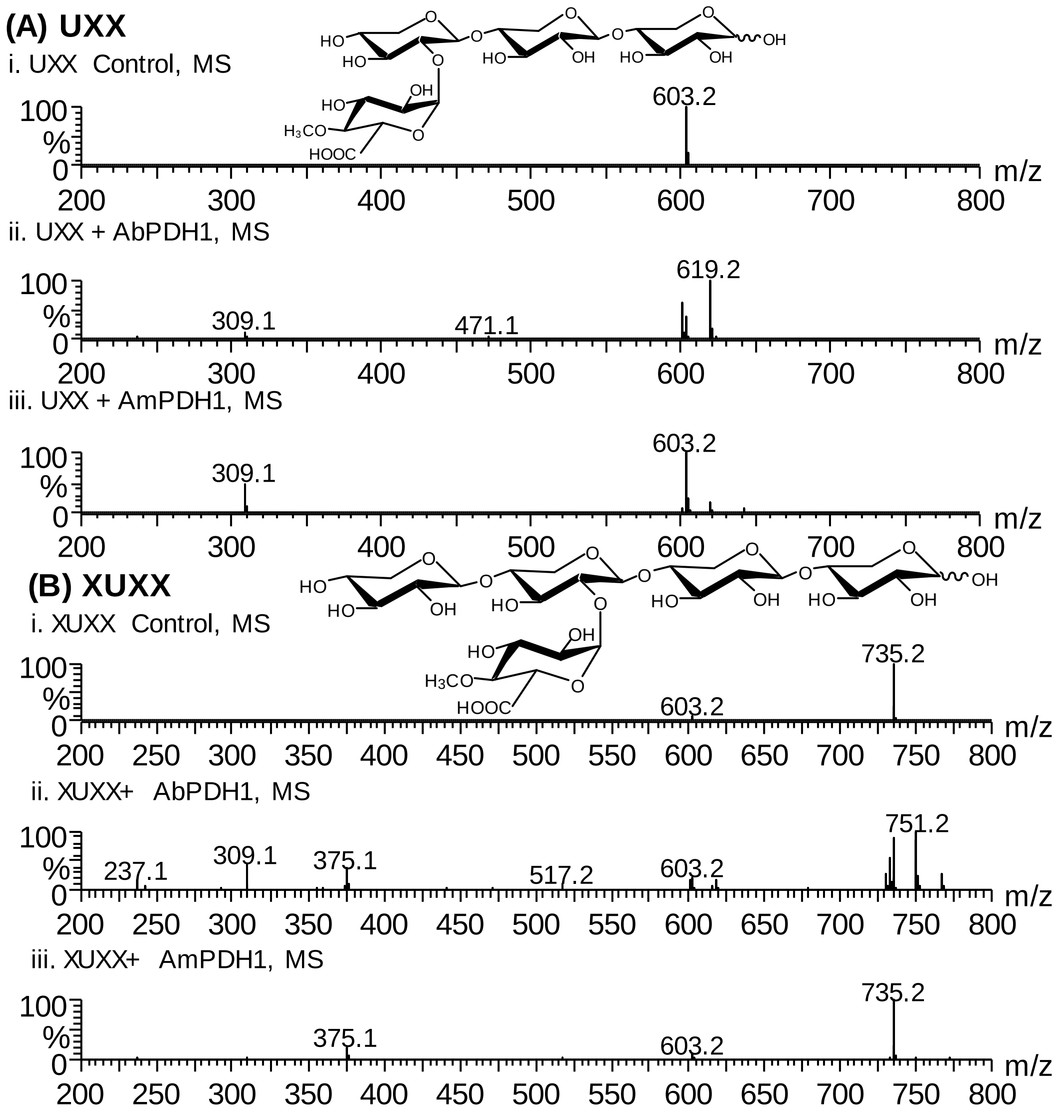

Figure S11. Negative ion MS spectra of (A) 2^3-(4-*O*-methyl-α-D-glucuronyl)-xylotriose (UXX) and (B) 2^3-(4-*O*-methyl-α-D-glucuronyl)-xylotetraose (XUXX). Control reactions, AbPDH1 oxidation, and AmPDH1 oxidation shown as i, ii, iii, respectively. Non-oxidized UXX and XUXX were ionized as anion with *m/z* of 603 and 751.

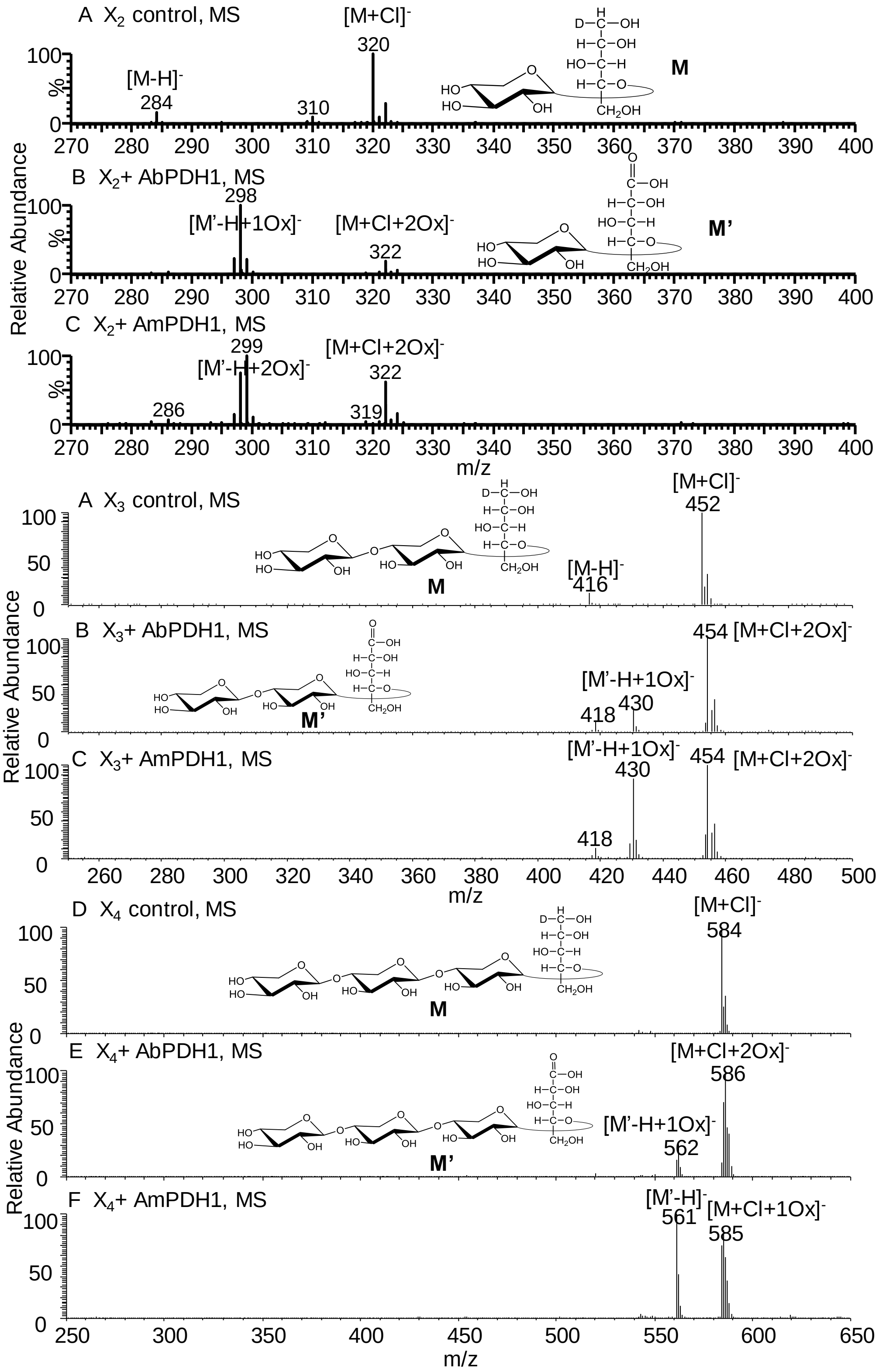

Figure S12. Negative MS spectra of $NaBD_4$ reduced linear xylooligosaccharides.

Figure S12. Continuing from previous page. (A) Xylobiose (X_2) after control reaction, (B) X_2 after reaction with AbPDH1, and (C) X_2 after reaction with AmPDH1. (D) Xylotriose (X_3) after control reaction, (E) X_3 after reaction with AbPDH1, and (F) X_3 after reaction with AmPDH1. (G) Xylotetraose (X_4) after control reaction, (H) X_4 after reaction with AbPDH1, (I) X_4 after reaction with AmPDH1. X_2 and its oxidized products were analyzed differently by Acquity Ultra High Performance Liquid Chromatography (UPLC) coupled to an ESI-Q-Tof mass spectrometer (Waters, MA. USA). In this case, a 30 µl sample from each reaction was mixed in 500 µl 50% ACN containing 0.1% ammonium hydroxide. A 7 µl aliquot was then injected onto a 1.7 µm, 2.1*100 mm Acquity UPLC BEH Amide (HILIC) column (Waters, MA, USA) coupled to the ESI-Q-Tof. The mobile phases were A) 80% ACN with 0.1% ammonium hydroxide and B) 20% ACN with 0.1% ammonium hydroxide. The elution gradient was as follows: from 80% ACN to 50% ACN in 10 min, back to 80% ACN in 0.1 min, and 12 min washing in 80% ACN. The flow rate was 250 µl/min and the oven temperature was set to 35 °C. The negative ion MS and MS/MS spectra of the X_2 samples were obtained, and corresponding chloride adduct ions were formed by adding 1 mg/ml ammonium chloride in 80% ACN post-column in a flow rate of 10 µl/min. The data collection time for the mass analyzer was set to 2-22 min to get rid of the non-volatile salt. The parameters for ESI-Q-Tof was set to the same as mentioned in section 2.6, except that the desolvation temperature was set to 400 °C. Collision Energy Ramp was used for fragmentation with initial energy at 10 V and final energy at 40 V.

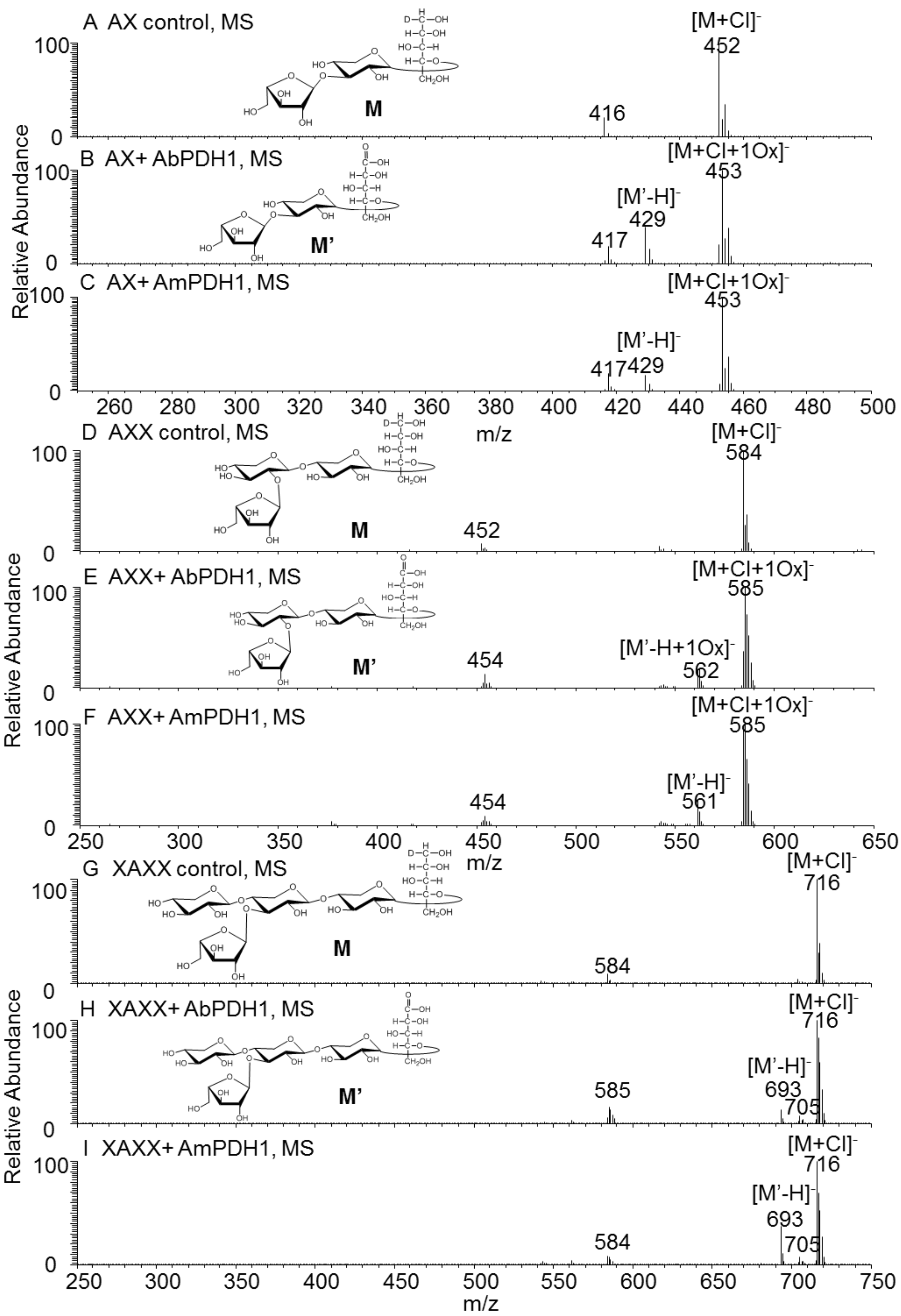

Figure S13. Negative MS spectra of $NaBD_4$ reduced Ara*f* substituted xylooligosaccharides.

Figure S13. Continuing from previous page. (A) 3^2-α-L-Arabinofuranosyl-xylobiose (AX) after control reaction, (B) AX after reaction with AbPDH1, and (C) AX after reaction with AmPDH1. (D) 2^3-α-L-Arabinofuranosyl-xylotriose (AXX) after control reaction, (E) AXX after reaction with AbPDH1, and (F) AXX after reaction with AmPDH1. (G) 3^3-α-L-Arabinofuranosyl-xylotetraose (XAXX) after control reaction, (H) XAXX after reaction with AbPDH1, and (I) XAXX after reaction with AmPDH1.

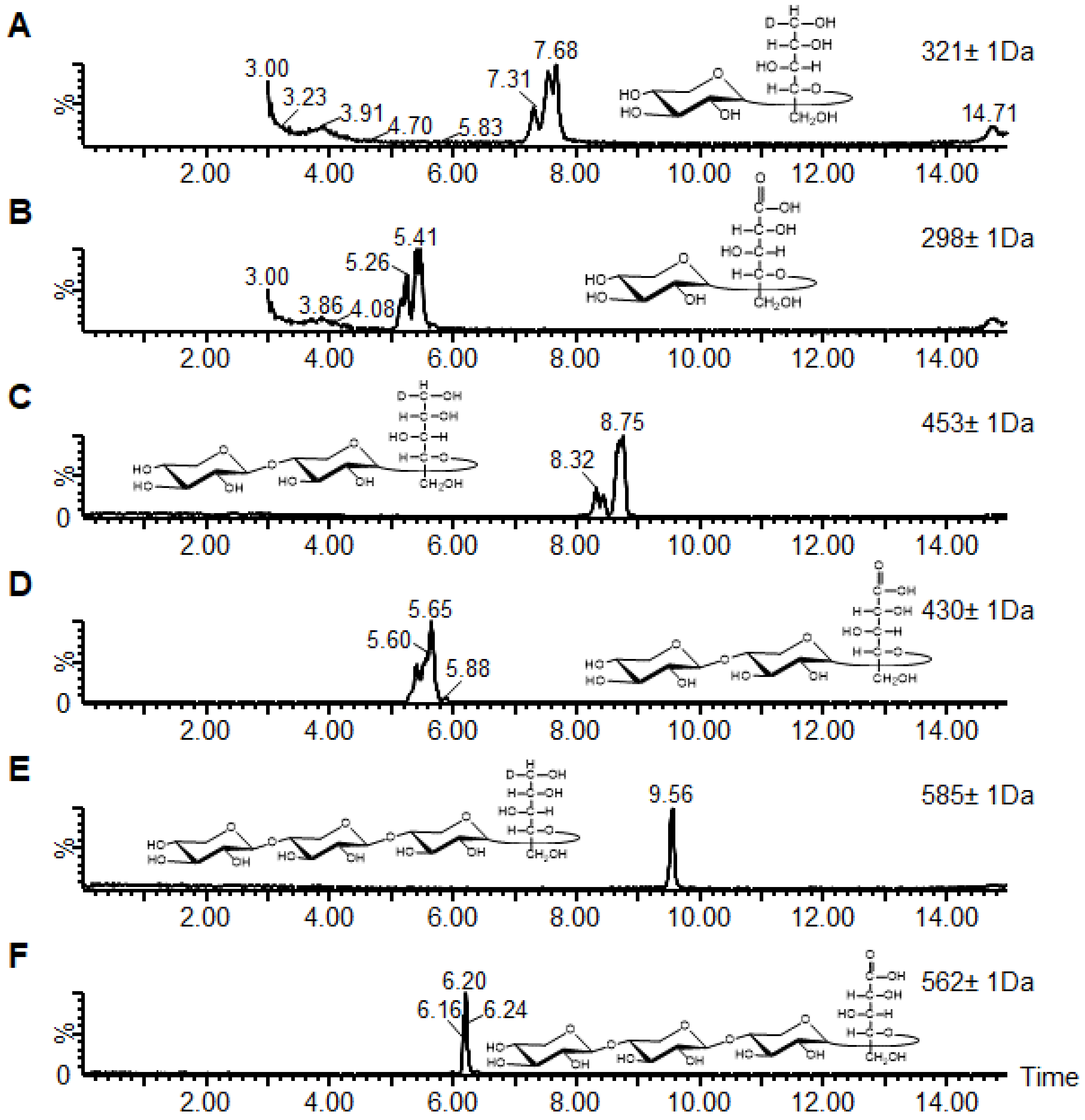

Figure S14. HILIC chromatogram (Single Ion Monitoring) of $NaBD_4$ reduced linear xylooligosaccharides. (A) Reduced neutral reaction products of oxidized xylobiose at m/z 321± 1Da, (B) reduced acidic reaction products of oxidized xylobiose at m/z 298± 1Da (C) reduced neutral reaction products of oxidized xylotriose at m/z 453± 1Da, (D) reduced acidic reaction products of oxidized xylotriose at m/z 430± 1Da, (E) reduced neutral reaction products of oxidized xylotetraose at m/z 585± 1Da, and (F) reduced acidic reaction products of oxidized xylotetraose at m/z 562± 1Da.

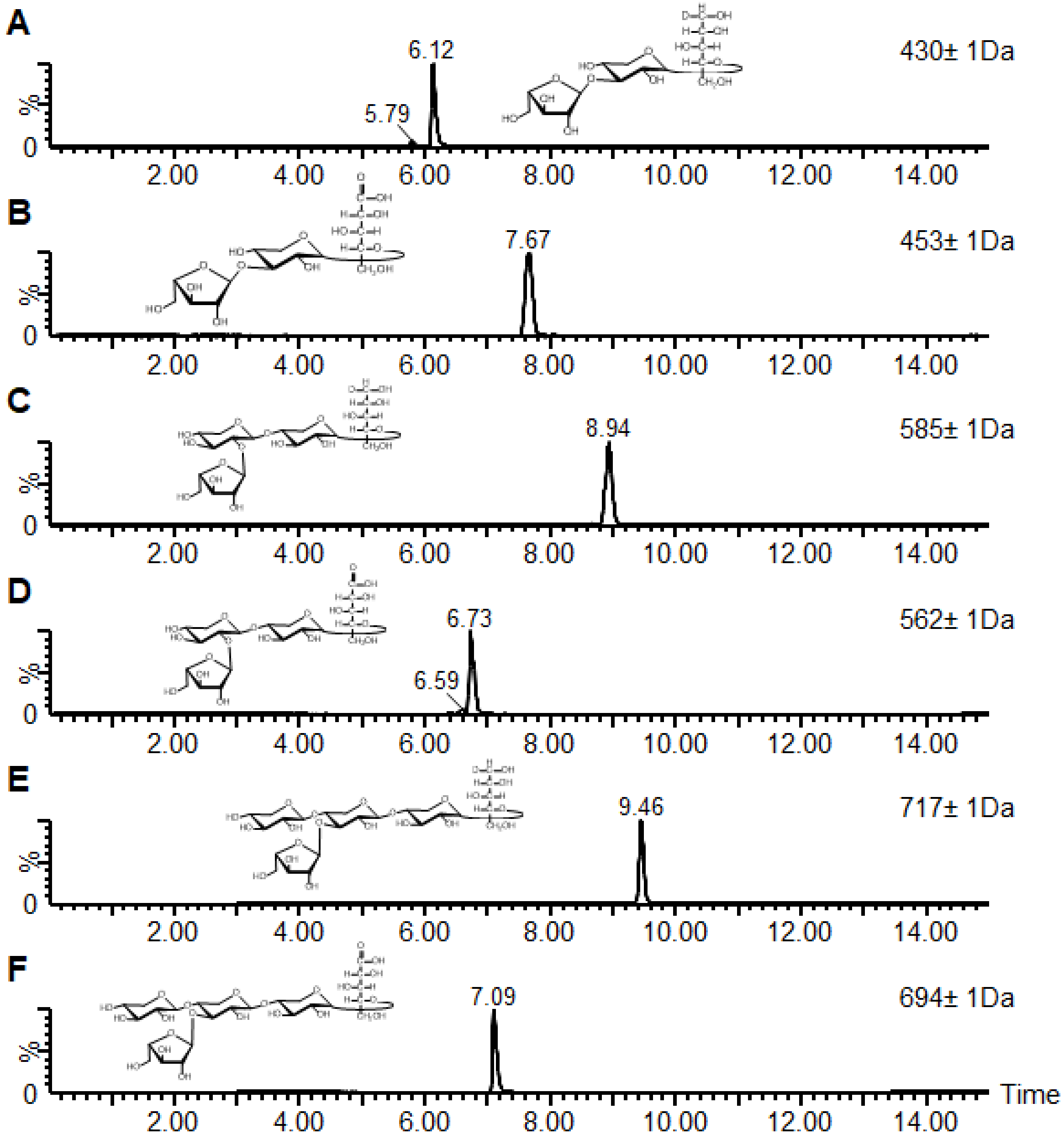

Figure S15. HILIC chromatogram (Single Ion Monitoring) of Ara*f* substituted xylooligosaccharides. (A) Reduced neutral reaction products of oxidized 3^2-α-L-arabinofuranosyl-xylobiose (A^3X) at m/z 453± 1Da, (B) reduced acidic reaction products of oxidized A^3X at m/z 430± 1Da, (C) reduced neutral reaction products of oxidized 2^3-α-L-arabinofuranosyl-xylotriose (A^2XX) at m/z 585± 1Da, (D) reduced acidic reaction products of oxidized A^2XX at m/z 562± 1Da, (E) reduced neutral reaction products of oxidized 3^3-α-L-Arabinofuranosyl-xylotetraose (XA^3XX) at m/z 717± 1Da, and (F) reduced acidic reaction products of oxidized XA^3XX at m/z 694± 1Da.

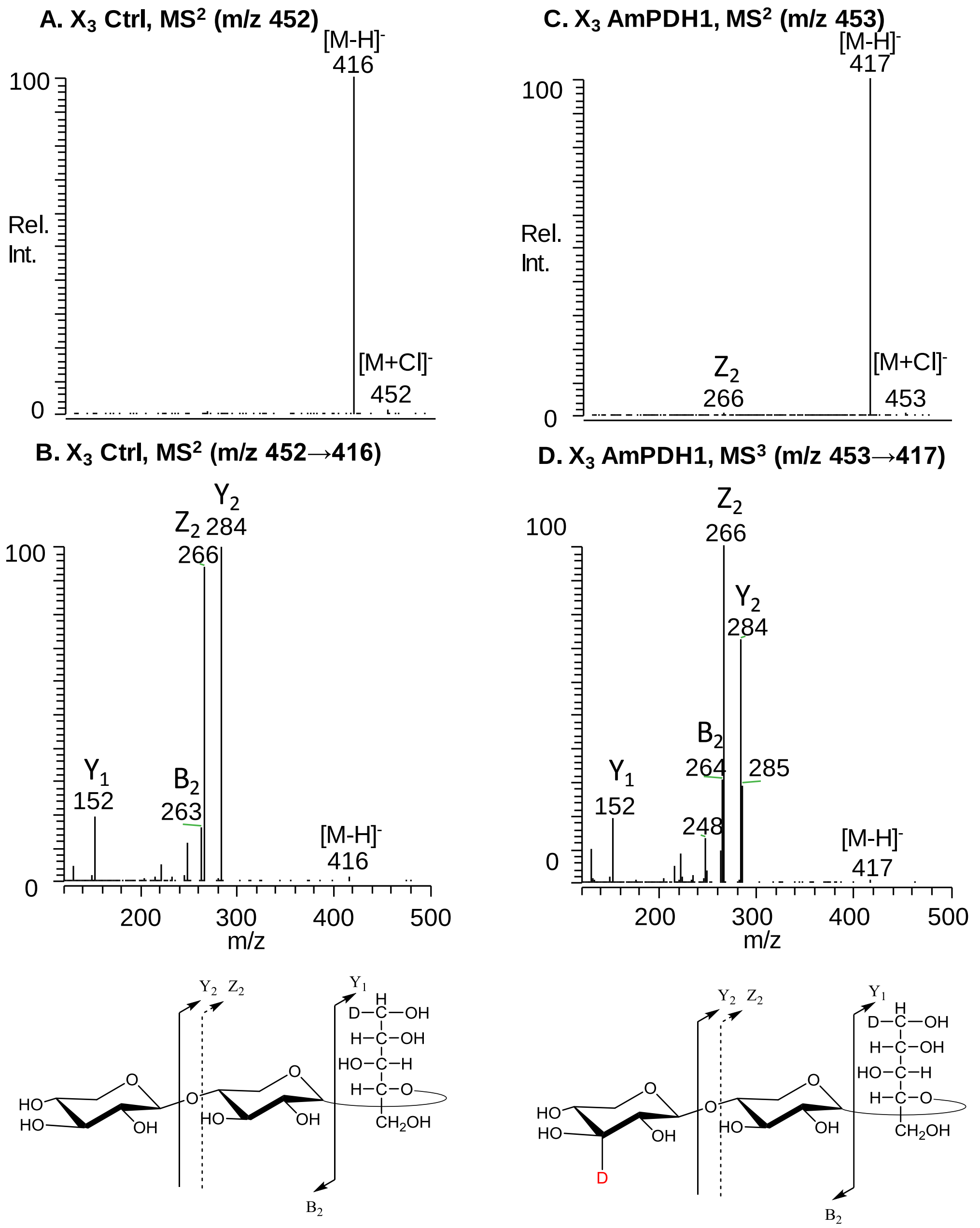

Figure S16. Extract of negative ion MS^n spectra of xylotriose (X_3) – Part One. Precursor ions are $[M+Cl]^-$ and product ions are deprotonated $[M-H]^-$. Reduced X_3 in control reaction: (A) MS^2 *m/z* 452, (B) MS^3 *m/z* 452→416. AmPDH1 treated X_3 with one oxidation at a secondary hydroxyl after $NaBD_4$ reduction: (C) MS^2 *m/z* 453 (D) MS^3 *m/z* 453→417. Product ions named according to (Domon and Costello, 1988) (The oxidation at C-2, C-3, and C-4 is not distinguishable).

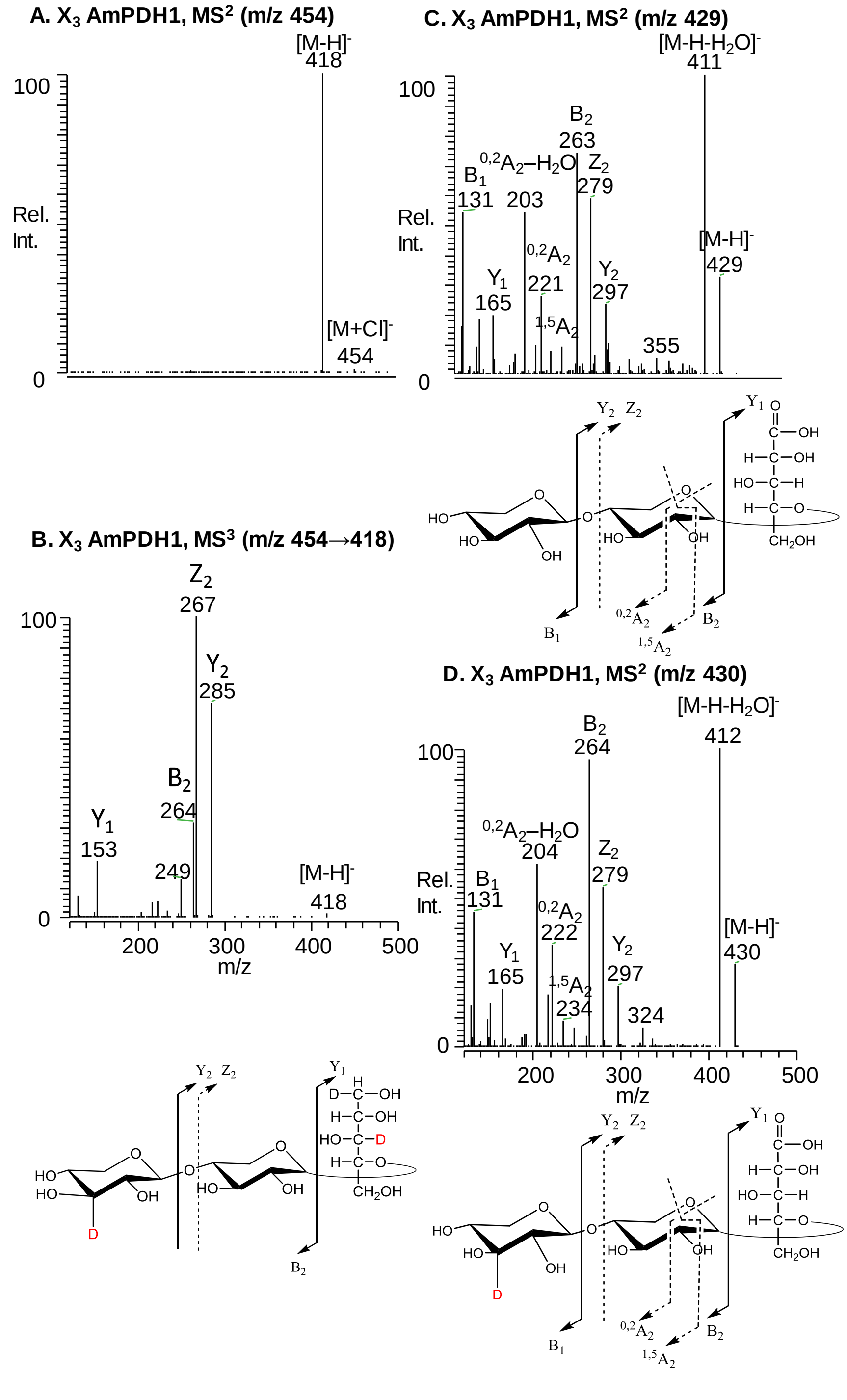

Figure S17. Extract of negative ion MS^n spectra of xylotriose (X_3) – Part Two. Precursor ions are $[M+Cl]^-$ (A) or $[M-H]^-$ (C, D) and product ions are deprotonated $[M-H]^-$. AmPDH1 treated X_3 with two oxidation at a secondary hydroxyl after $NaBD_4$ reduction: (A) MS^2 *m/z* 454, (B) MS^3 *m/z* 454→418. AmPDH1 treated X_3 with one carboxyl acid after $NaBD_4$ reduction: (C) MS^2 *m/z* 429. AmPDH1 treated X_3 with one ketone and one carboxyl acid after $NaBD_4$ reduction: (D) MS^2 m/z 430. Product ions named according to (Domon and Costello, 1988). Oxidation at C-2, C-3, and C-4 is not distinguishable.

References

Domon, B., and Costello, C. E. (1988). A systematic nomenclature for carbohydrate fragmentations in FAB-MS/MS spectra of glycoconjugates. *Glycoconj. J.* 5, 397–409. doi:10.1007/BF01049915.

Gonaus, C., Kittl, R., Sygmund, C., Haltrich, D., and Peterbauer, C. (2016). Transcription analysis of pyranose dehydrogenase from the basidiomycete *Agaricus bisporus* and characterization of the recombinantly expressed enzyme. *Protein Expr. Purif.* 119, 36–44. doi:10.1016/j.pep.2015.11.003.

Graf, M. M. H., Weber, S., Kracher, D., Kittl, R., Sygmund, C., Ludwig, R., et al. (2017). Characterization of three pyranose dehydrogenase isoforms from the litter-decomposing basidiomycete *Leucoagaricus meleagris* (syn. *Agaricus meleagris*). *Appl. Microbiol. Biotechnol.* 101, 2879–2891. doi:10.1007/s00253-016-8051-1.

Krondorfer, I., Brugger, D., Paukner, R., Scheiblbrandner, S., Pirker, K. F., Hofbauer, S., et al. (2014). *Agaricus meleagris* pyranose dehydrogenase: Influence of covalent FAD linkage on catalysis and stability. *Arch. Biochem. Biophys.* 558, 111–119. doi:10.1016/j.abb.2014.07.008.

Kujawa, M., Volc, J., Halada, P., Sedmera, P., Divne, C., Sygmund, C., et al. (2007). Properties of pyranose dehydrogenase purified from the litter-degrading fungus *Agaricus xanthoderma*. *FEBS J.* 274, 879–894. doi:10.1111/j.1742-4658.2007.05634.x.

Staudigl, P., Krondorfer, I., Haltrich, D., and Peterbauer, C. K. (2013). Pyranose dehydrogenase from *Agaricus campestris* and *Agaricus xanthoderma*: Characterization and applications in carbohydrate conversions. *Biomolecules* 3, 535–552. doi:10.3390/biom3030535.

Sygmund, C., Gutmann, A., Krondorfer, I., Kujawa, M., Glieder, A., Pscheidt, B., et al. (2012). Simple and efficient expression of *Agaricus meleagris* pyranose dehydrogenase in *Pichia pastoris*. *Appl. Microbiol. Biotechnol.* 94, 695–704. doi:10.1007/s00253-011-3667-7.

Sygmund, C., Kittl, R., Volc, J., Halada, P., Kubátová, E., Haltrich, D., et al. (2008). Characterization of pyranose dehydrogenase from *Agaricus meleagris* and its application in the C-2 specific conversion of D-galactose. *J. Biotechnol.* 133, 334–342. doi:10.1016/j.jbiotec.2007.10.013.

Pisanelli, I., Kujawa, M., Gschnitzer, D., Spadiut, O., Seiboth, B., and Peterbauer, C. (2010). Heterologous expression of an *Agaricus meleagris* pyranose dehydrogenase-encoding gene in *Aspergillus* spp. and characterization of the recombinant enzyme. *Appl. Microbiol. Biotechnol.* 86, 599–606. doi:10.1007/s00253-009-2308-x.

Volc, J., Kubátová, E., Wood, D. A., and Daniel, G. (1997). Pyranose 2-dehydrogenase, a novel sugar oxidoreductase from the basidiomycete fungus *Agaricus bisporus*. *Arch. Microbiol.* 167, 119–125. doi:10.1007/s002030050424.
